# Degeneration of structural brain networks is associated with cognitive decline after ischaemic stroke

**DOI:** 10.1093/braincomms/fcaa155

**Published:** 2020-09-26

**Authors:** Michele Veldsman, Hsiao-Ju Cheng, Fang Ji, Emilio Werden, Mohamed Salah Khlif, Kwun Kei Ng, Joseph K W Lim, Xing Qian, Haoyong Yu, Juan Helen Zhou, Amy Brodtmann

**Affiliations:** Department of Experimental Psychology, Wellcome Centre for Integrative Neuroimaging, University of Oxford, Oxford, UK; The Florey Institute of Neuroscience and Mental Health, University of Melbourne, Melbourne, Australia; Department of Medicine, Center for Sleep and Cognition, Yong Loo Lin School of Medicine, National University of Singapore, Singapore, Singapore; Centre for Cognitive Neuroscience, Neuroscience and Behavioural Disorders Program, Duke-NUS Medical School, Singapore, Singapore; Department of Biomedical Engineering, Faculty of Engineering, National University of Singapore, Singapore, Singapore; Department of Medicine, Center for Sleep and Cognition, Yong Loo Lin School of Medicine, National University of Singapore, Singapore, Singapore; Centre for Cognitive Neuroscience, Neuroscience and Behavioural Disorders Program, Duke-NUS Medical School, Singapore, Singapore; The Florey Institute of Neuroscience and Mental Health, University of Melbourne, Melbourne, Australia; The Florey Institute of Neuroscience and Mental Health, University of Melbourne, Melbourne, Australia; Department of Medicine, Center for Sleep and Cognition, Yong Loo Lin School of Medicine, National University of Singapore, Singapore, Singapore; Centre for Cognitive Neuroscience, Neuroscience and Behavioural Disorders Program, Duke-NUS Medical School, Singapore, Singapore; Department of Medicine, Center for Sleep and Cognition, Yong Loo Lin School of Medicine, National University of Singapore, Singapore, Singapore; Centre for Cognitive Neuroscience, Neuroscience and Behavioural Disorders Program, Duke-NUS Medical School, Singapore, Singapore; Department of Medicine, Center for Sleep and Cognition, Yong Loo Lin School of Medicine, National University of Singapore, Singapore, Singapore; Centre for Cognitive Neuroscience, Neuroscience and Behavioural Disorders Program, Duke-NUS Medical School, Singapore, Singapore; Department of Biomedical Engineering, Faculty of Engineering, National University of Singapore, Singapore, Singapore; NUS Graduate School for Integrative Sciences and Engineering, National University of Singapore, Singapore, Singapore; Department of Medicine, Center for Sleep and Cognition, Yong Loo Lin School of Medicine, National University of Singapore, Singapore, Singapore; Centre for Cognitive Neuroscience, Neuroscience and Behavioural Disorders Program, Duke-NUS Medical School, Singapore, Singapore; NUS Graduate School for Integrative Sciences and Engineering, National University of Singapore, Singapore, Singapore; Center for Translational Magnetic Resonance Research, Yong Loo Lin School of Medicine National University of Singapore, Singapore, Singapore; The Florey Institute of Neuroscience and Mental Health, University of Melbourne, Melbourne, Australia

**Keywords:** stroke, structural networks, cognition, neurodegeneration

## Abstract

Over one-third of stroke patients has long-term cognitive impairment. The likelihood of cognitive dysfunction is poorly predicted by the location or size of the infarct. The macro-scale damage caused by ischaemic stroke is relatively localized, but the effects of stroke occur across the brain. Structural covariance networks represent voxelwise correlations in cortical morphometry. Atrophy and topographical changes within such distributed brain structural networks may contribute to cognitive decline after ischaemic stroke, but this has not been thoroughly investigated. We examined longitudinal changes in structural covariance networks in stroke patients and their relationship to domain-specific cognitive decline. Seventy-three patients (mean age, 67.41 years; SD = 12.13) were scanned with high-resolution magnetic resonance imaging at sub-acute (3 months) and chronic (1 year) timepoints after ischaemic stroke. Patients underwent a number of neuropsychological tests, assessing five cognitive domains including attention, executive function, language, memory and visuospatial function at each timepoint. Individual-level structural covariance network scores were derived from the sub-acute grey-matter probabilistic maps or changes in grey-matter probability maps from sub-acute to chronic using data-driven partial least squares method seeding at major nodes in six canonical high-order cognitive brain networks (i.e. dorsal attention, executive control, salience, default mode, language-related and memory networks). We then investigated co-varying patterns between structural covariance network scores within canonical distributed brain networks and domain-specific cognitive performance after ischaemic stroke, both cross-sectionally and longitudinally, using multivariate behavioural partial least squares correlation approach. We tested our models in an independent validation data set with matched imaging and behavioural testing and using split-half validation. We found that distributed degeneration in higher-order cognitive networks was associated with attention, executive function, language, memory and visuospatial function impairment in sub-acute stroke. From the sub-acute to the chronic timepoint, longitudinal structural co-varying patterns mirrored the baseline structural covariance networks, suggesting synchronized grey-matter volume decline occurred within established networks over time. The greatest changes, in terms of extent of distributed spatial co-varying patterns, were in the default mode and dorsal attention networks, whereas the rest were more focal. Importantly, faster degradation in these major cognitive structural covariance networks was associated with greater decline in attention, memory and language domains frequently impaired after stroke. Our findings suggest that sub-acute ischaemic stroke is associated with widespread degeneration of higher-order structural brain networks and degradation of these structural brain networks may contribute to longitudinal domain-specific cognitive dysfunction.

## Teaser

Examining network-based degeneration, Veldsman and Cheng et al. show that higher-order structural covariance networks are associated with cognition in sub-acute stroke and that greater degeneration of these networks is associated with longitudinal cognitive decline after ischaemic stroke.

## Graphical Abstract

**Graphical Abstract:**
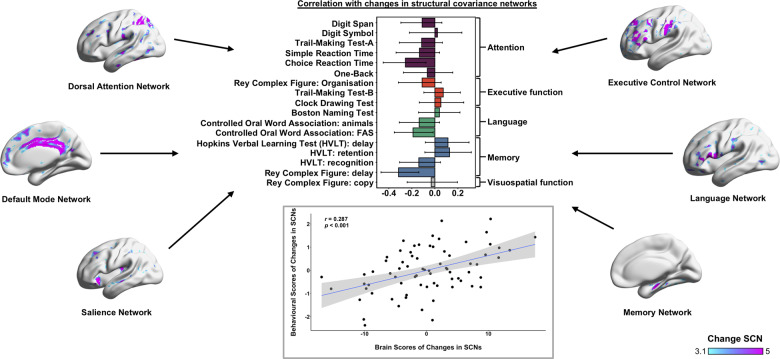

## Introduction

Cognitive decline is common after ischaemic stroke (Hochstenbach *et al.*, 1998; Levine *et al.*, 2015) and is associated with poor quality of life for stroke patients (Cumming *et al.*, 2014). Cognitive decline after stroke may be global in nature, or may be associated with one or more cognitive domains, such as memory, attention or language. Surprisingly, infarct location does not closely predict the severity of the post-stroke cognitive deficit (Fox, 2018). Infarcts in different regions of the brain can result in similar cognitive deficits, whereas infarcts in the same region can result in different profiles of cognitive impairments (Fox, 2018). Since no single brain region works in isolation, it stands to reason that an infarct will have distributed consequences, affecting incoming and outgoing connections to the damaged area (Grefkes and Fink, 2011, 2014). This disruption appears to be both in terms of brain function (Siegel *et al.*, 2016) and in terms of white-matter (WM) connectivity (Stinear *et al.*, 2007), which have been studied extensively in stroke (Grefkes and Fink, 2014). The impact of stroke on structural covariance networks (SCNs) has not been thoroughly investigated, despite evidence of atrophy after stroke that is remote from the infarct location (Firbank *et al.*, 2007; Werden *et al.*, 2017; Veldsman *et al.*, 2018). Structural covariance networks are constructed based on shared inter-regional morphological characteristics, such as grey-matter (GM) volume or cortical thickness that are estimated across populations (Evans, 2013). These SCNs closely mirror intrinsic functional networks and have aided understanding of a diverse range of neurological diseases, including epilepsy, schizophrenia and Alzheimer’s disease (Evans, 2013).

Brain atrophy is a normal part of healthy aging, but it is accelerated in the presence of dementia causing pathologies (Spreng and Turner, 2013) and cerebrovascular burden (Veldsman, 2017). Across dementia sub-types, the pattern of brain atrophy mirrors the healthy structural and functional networks responsible for the dominantly impaired function (Seeley *et al.*, 2009; Zhou *et al.*, 2010; Zhou *et al.*, 2012; Zhou and Seeley, 2014). For example, language networks show widespread atrophy in patients with primary progressive aphasia where language is the dominant deficit. The integrity of the networks underlying different cognitive functions may be critical for the preserved functioning of cognitive domains after stroke.

We examined structural covariance in our discovery data set in canonical higher-order cognitive brain networks in 73 stroke patients 3 months and 1 year after ischaemic stroke. We predicted that the integrity of SCNs at 3-month post-stroke would reflect cognitive performance. If longitudinal changes in SCNs were to be associated with longitudinal cognitive decline, the relationship at the sub-acute phase is unlikely to be epiphenomenal. Therefore, to further test our hypothesis, we investigated whether changes in SCNs from 3-month to 1-year post-stroke would be associated with changes in cognitive function. We tested our models in our validation data set, an independent data set with matched imaging and neuropsychological and cognitive testing and performed split-half analysis for longitudinal validation.

## Materials and methods

### Participants

Stroke patient data from the Cognition and Neocortical Volume after Stroke study (Brodtmann *et al.*, 2014) were used in this study. Our discovery data set included data from 80 stroke patients who underwent 3-Tesla magnetic resonance imaging (MRI) and detailed neuropsychological tests at the 3-month and 1-year timepoints. Here, we refer to the 3-month timepoint as sub-acute and the 12-month timepoint as chronic. Three months is the most commonly used timepoint for assessing outcomes after stroke. Among 80 participants, three participants were excluded due to excessive head movement during brain scans and four participants were excluded based on the lack of image homogeneity evaluated by mean correlation after voxel-based morphometry (VBM). The remaining 73 participants (mean age, 67.41 years; SD = 12.13, Table 1) were included in the analysis. Our validation data set was made up of data from 26 patients from the same cohort scanned at a later date with the same scanner, MRI sequences and neuropsychological and cognitive testing. Matched quality control to our discovery data set excluded three patients on the basis of excessive movement in their sub-acute scans. One patient lacked all cognitive data and was excluded on this basis; this left a total of 22 patients (mean age, 68.77 years; SD = 9.65; Supplementary Materials Section 6 and Supplementary Table 3). All participants gave written informed consent for the study that was approved by local hospital ethics committees in line with the Declaration of Helsinki.

**Table 1:** Participant demographic and behavioural characteristics

Demographics	Statistics
Age (years), mean (SD)	67.41 (12.13)
Sex (male/female)	51/22
Handedness (left/right)	6/67
Education (years), mean (SD)	12.89 (3.75)
Infarct volume (mm^3^), mean (SD)	5786.62 (9316.65)
NIHSS on admission, median (25th, 75th percentile)	2 (1,4)
NIHSS at 3 months, median (25th, 75th percentile)	0 (0,2)
mRS on admission, median (25th, 75th percentile)	1 (1,2)
mRS at 3 months, median (25th, 75th percentile)	1 (1,2)
MoCA, mean (SD)	24.26 (3.43)
Scan interval (days), mean (SD)	276.92 (26.14)

MoCA = Montreal Cognitive Assessment; mRS = modified Rankin Score; NIHSS = National Institute of Health Stroke Scale; SD = standard deviation.

### Neuropsychological testing

Neuropsychological and cognitive tests were administered at each timepoint, as outlined in the published protocol (Brodtmann *et al.*, 2014) and detailed in Supplementary Materials Section 1 and Supplementary Table 1 (including percentage of missing data for each test at each timepoint; <5% missing in any cognitive domain). Missing data were imputed using the Missing Data Imputation (MDI) Toolbox for MATLAB (Folch-Fortuny *et al.*, 2016, 2017). Briefly, missing data were first replaced by the mean of their corresponding test scores and partial least squares (PLS) was used to build the statistical model with a response matrix. The original test score matrix and the response matrix were auto-scaled to fit a PLS model to predict the missing values. Auto-scaling and model fitting were iterated until convergence was reached.

### Image acquisition

Participants were scanned on a 3-Tesla Siemens Tim Trio scanner using a 12-channel head coil (Erlangen, Germany). The scan protocol comprised a high-resolution magnetization prepared rapid gradient recalled echo sequence [repetition time (TR) = 1900 ms, echo time (TE) = 2.55 ms, inversion time =900 ms, flip angle = 9°, 160 sagittal slices, matrix size = 256 × 256, voxel size = 1 mm isotropic] and a 3D SPACE-fluid attenuated inversion recovery (FLAIR) sequence (TR = 6000 ms, TE = 380 ms, inversion time = 2100 ms, flip angle = 120°, 160 axial slices, matrix size = 256 × 256, voxel size =1 mm isotropic).

### Image processing

**Figure 1:**
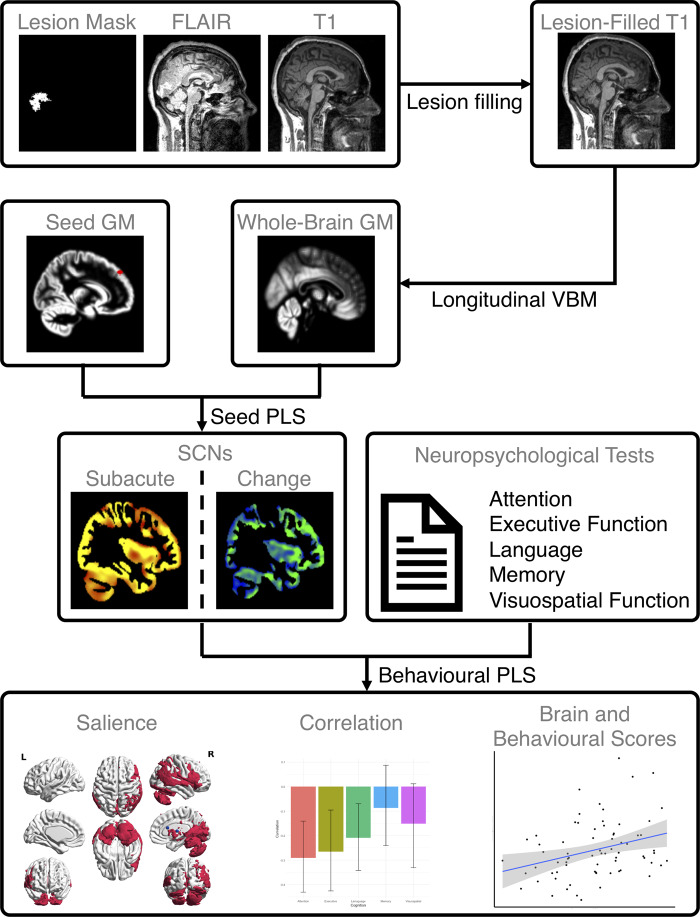
**Overview of study design schematic.** For each participant, we first used a Lesion Segmentation Tool (LST) (Schmidt *et al.*, 2012) with a lesion mask as well as FLAIR and T_1_-weighted images to generate a lesion-filled T_1_-weighted image for each participant. Then, we applied the longitudinal VBM pipeline using a computational anatomy toolbox (CAT12) to obtain smoothed and normalized GM images. With pre-defined regions of interest based on canonical brain networks, we extracted the mean GM volume of each seed. Seed PLS was used to co-vary the seed and whole-brain GM for deriving the SCNs at 3-month post-stroke (sub-acute timepoint) and change from 3-month to 1-year (chronic timepoint) post-stroke, respectively, for all participants. Finally, SCN scores and neuropsychological test scores at the sub-acute timepoint were input into a behavioural PLS model to examine the covariance between SCNs and cognition for all participants. The SCN change scores and changes of neuropsychological test scores were input into another behavioural PLS model to investigate the cognitive decline over time based on SCN degradation from 3-month to 1-year post-stroke for all participants. Abbreviations: FLAIR = fluid-attenuated inversion recovery; GM = grey matter; PLS = partial least squares; SCN = structural covariance network; VBM = voxel-based morphometry.

Infarcts were manually traced on the FLAIR image and verified by a stroke neurologist (A.B.). Infarcts were converted into a binary lesion map (see Supplementary Materials Section 3 and Supplementary Fig. 1 for the group lesion overlap map). The individual FLAIR image, T_1_-weighted image and binarized lesion maps were used as inputs to perform lesion filling to correct the intensity of the lesions using the Lesion Segmentation Tool (LST) (Schmidt *et al.*, 2012) for Statistical Parametric Mapping (SPM12; Wellcome Trust Centre for Neuroimaging). The LST creates a lesion probability map from the GM, WM and cerebrospinal fluid (CSF)-segmented T_1_-weighted image. The intensity distribution is calculated using the FLAIR image based on these three tissue classes. The resultant lesion probability map combined with a binarized lesion map was used to fill lesions based on local information, to increase the accuracy of lesion filling. After lesion filling, we then applied the longitudinal VBM pipeline using a computational anatomy toolbox (CAT12) for SPM12 on the lesion-filled T_1_-weighted images. Subject-level GM probability maps were obtained from T_1_-weighted images by following our previously published approach (Ng *et al.*, 2016), including (i) inter-participant image realignment across timepoints and intra-participant signal inhomogeneity correction for each participant to create a mean reference image for each subject; (ii) segmentation of the bias-corrected and reference images into GM, WM and CSF using an Adaptive Maximum A Posterior technique (Rajapakse *et al.*, 1997); (iii) an initial affine registration applied to the bias-corrected image to improve the initial SPM segmentation; (iv) non-linear Diffeomorphic Anatomical Registration Through Exponentiated Lie Algebra registration (Ashburner, 2007) from all subjects to create a study-specific template in MNI space; (v) spatial normalization of each segmented GM/WM reference image and individual GM/WM probability maps to the customized template in MNI space; (vi) modulation by multiplying voxel values with the linear and non-linear component of the Jacobian determinant; (vii) smoothing on the normalized GM and WM probability maps with an isotropic 8-mm Gaussian kernel. An overview of the preprocessing and structural covariance pipeline is shown in Fig. 1.

### Structural covariance network analysis

#### Region of interest derivation

Six canonical brain networks were selected corresponding to cognitive domains tested in this study, including the dorsal attention (DAN), executive control (ECN), salience (SN), default mode (DMN), language-related (LN) and memory (MN) networks. For each network, two ROIs were chosen from the peak foci reported in the previous studies (Supplementary Table 2; Fig. 1). We selected seed regions for each network on the basis that they have been shown to reliably produce the relevant network across a range of methods and imaging modalities. In particular, we selected networks that have been shown to be related to the associated cognitive domain tested here and have been shown to be mirrored within SCNs. The seed for the dorsal attention network represents the peak region derived from a meta-analysis of attention-related tasks (Corbetta and Shulman, 2002). We chose this region because it is reliably activated in response to the top-down directed, voluntary deployment of attention as required by the behavioural attention tasks in our cognitive battery (Corbetta and Shulman, 2002; Fox *et al.*, 2006). Furthermore, the network shows correlated spontaneous activity even in the absence of a task (Fox *et al.*, 2006; Vossel *et al.*, 2014). The seeds for the salience and executive control networks were derived from Seeley *et al.* (2007). The seeds represent the peak regions of activation from executive control tasks and tasks requiring personal salience or interoceptive processing (Seeley *et al.*, 2007). The resultant dissociable networks can be reliably derived from task-based functional MRI, seed-based and independent component analysis of resting-state MRI (Seeley *et al.*, 2007). Importantly, these networks are also mirrored in SCNs which show syndrome specific atrophy across dementia sub-types (Seeley *et al.*, 2009; Zhou *et al.*, 2010). Language network seeds were selected in a similar way, from the peak regions in a language network derived in healthy controls that was mirrored as a SCN and that showed atrophy in language-impaired dementia syndromes (Seeley *et al.*, 2009; Zielinski *et al.*, 2010). For the default mode network, seeds that have been used previously in the derivation of task-based (Sridharan *et al.*, 2008), resting-state (Greicius *et al.*, 2003; Chong *et al.*, 2017) and SCNs (Zhou *et al.*, 2012) were used. Finally, ROIs for the memory network were derived from peak regions in memory task-based fMRI (Koechlin *et al.*, 1999) that also produces the hippocampal cortical network when used in seed-based resting state functional connectivity analysis (Vincent *et al.*, 2008).

We quantified the degree of overlap between the group lesion map and a conjunction map of all the ROI seeds (Supplementary Materials Section 3 and Supplementary Fig. 1) to ensure that our results were not driven by damage within the seed regions. Spherical ROIs of 4 mm radius were used to extract the mean GM volume from the pre-processed GM probability maps using MarsBaR region of interest toolbox for SPM12. Notably, for the change of GM volume, we subtracted the GM probability maps at the sub-acute timepoint from the maps at the chronic timepoint for each subject and the resultant GM probability maps were used to extract mean change of GM volume with 12 4-mm radius spherical ROIs.

#### Structural covariance network derivation

Seed PLS was used to estimate structural covariance of each canonical brain network (McIntosh and Lobaugh, 2004). Open-source PLS software written in MATLAB (https://www.rotman-baycrest.on.ca/index.php?section=84; 28 September 2020, date last accessed) (Krishnan *et al.*, 2011) was used for all PLS analyses. The input to the seed PLS was the extracted mean GM volume of each seed and the pre-processed, whole-brain GM images (thresholded at signal intensity of 0.45). To derive the co-varying pattern between the seed GM volume and the rest of the brain, we performed singular value decomposition on the mean-centred and normalized input (Eckart and Young, 1936), a matrix in which rows correspond to participants and columns correspond to brain GM voxels. As such, structural covariance is estimated on a group level in order to show dominant co-variation patterns in morphology across the brain. The co-variation pattern, formally known as latent variables (LVs), carries two key pieces of information. The first one is a voxel-wise ‘brain salience’ 3D volume that shows the structural co-variation pattern. Voxels showing higher salience value have stronger associations with the seed GM; that is, the core regions in the SCN. The second one is a set of summary scores, called ‘brain scores’, which can be used to make individual level inferences. Briefly, a brain score for each participant was derived by multiplying the whole-brain GM images and the right singular matrix (i.e. brain salience), serving as an estimate of individual-level covariance. Thus, participants whose GM morphology showed higher resemblance to the group-level structural covariance had higher brain scores. The statistical significance of an LV was evaluated using a permutation test (i.e. if the covariance accounted for by the seed GM exceeded what could be obtained by chance, estimated by randomly permuting the input matrix rows 5000 times). The stability of each voxel in the brain salience of the LV was quantified using a bootstrap ratio, calculated by dividing the voxel salience value by its standard error (i.e. akin to a *Z*-score), estimated by bootstrapping (re-sampling of input matrix rows 1000 times). Voxels with high bootstrap ratios contribute most to the brain co-variation pattern.

To control for potential influences of confounding variables, separate linear regression analyses were performed on the brain scores of each seed for the sub-acute timepoint and longitudinal change. For the sub-acute timepoint, the variable of interest was the brain score of the seed. The confounding variables were age, sex, handedness, log-transformed infarct volume and total intra-cranial volume. For change, the scan interval was input as an additional confounding variable. The unstandardized residual brain scores of 12 seeds, referred to as the SCN scores, were used for further behavioural PLS analysis.

To ensure that the results were not driven by global atrophy, we also tested the discovery and validation models, replacing total intra-cranial volume with GM volume or total brain volume (GM and WM volume) normalized by total intra-cranial volume (Supplementary Materials Section 5). For the longitudinal analyses, we additionally controlled for change in global atrophy (chronic−sub-acute global atrophy; see Supplementary Materials Section 5.2).

### Statistical analysis

#### Behavioural PLS analysis

Similar to the seed PLS, behavioural PLS was performed to investigate the multivariate relationships between the neuropsychological test scores and the SCN scores. The neuropsychological test scores were unstandardized residuals after linear regression to control for confounding variables. The dependent variable was the score of each neuropsychological test and the confounding variables were age, sex, handedness, log-transformed infarct volume and total intra-cranial volume. Similarly, the scan interval was input as an additional confounding variable in the longitudinal analysis. The behavioural PLS model examined the 17 neuropsychological test scores and 12 network-specific SCN scores at the sub-acute timepoint. The behavioural score was also derived by multiplying neuropsychological test scores and behavioural salience to estimate the test-dependent differences in the SCN–cognition correlation. Identical to the SCN only analysis, statistical significance and the importance and reliability of each test score in the SCN–cognition relationship were evaluated with permutation tests based on 5000 repetitions and bootstrapping based on 1000 repetitions, respectively.

A second behavioural PLS model was built to study the relationship between changes in neuropsychological test scores and changes in SCNs from sub-acute (3 months) to chronic (1 year) stroke after the procedure outlined in the first behavioural PLS model. Changes in 17 neuropsychological test scores and 12 network-specific SCN change scores were input into this second behavioural PLS model with the same permutation and bootstrapping procedures.

Two additional infarct volume control analyses for the sub-acute timepoint and change from sub-acute to chronic timepoint were performed without controlling for the infarct volume (Supplementary Materials Section 4).

The independent validation data set was processed in exactly the same way, without any exceptions, at the sub-acute timepoint (Supplementary Materials Section 5). Due to a low sample size at the chronic timepoint, we used split-half validation, repeated five times with random sampling (Supplementary Materials Section 6.2).

### Data availability

The data that support the findings of this study are available from the corresponding author upon reasonable request.

## Results

### Relationship between SCNs and cognitive function in sub-acute stroke

**Figure 2:**
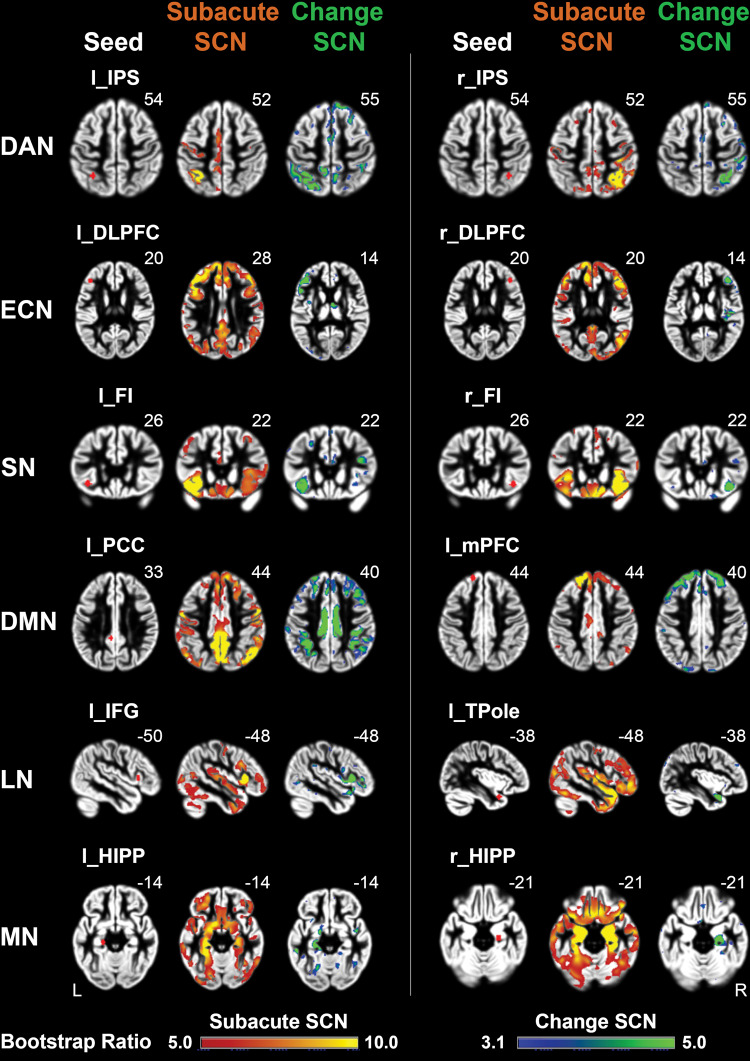
**Seed regions of canonical brain networks and structural covariance networks in sub-acute and chronic stroke patients.** For six canonical brain networks, two seed regions (red dot, first column) were selected for investigating the co-varying patterns of SCNs. The derived SCNs at the sub-acute timepoint (second column in orange) and for change from sub-acute to chronic stroke (third column in green). Abbreviations: DAN = dorsal attention network; DMN = default mode network; ECN = executive control network; l_DLPFC = left dorsolateral prefrontal cortex; l_FI = left frontal insula; l_HIPP = left hippocampus; l_IFG = left inferior frontal gyrus; l_IPS = left intra-parietal sulcus; l_mPFC = left medial prefrontal cortex; l_PCC = left posterior cingulate cortex; l_TPole = left temporal pole; LN = language-related network; MN = memory network; r_DLPFC = right dorsolateral prefrontal cortex; r_HIPP = right hippocampus; r_FI = right frontal insula; r_IPS = right intra-parietal sulcus; SCN = structural covariance network; SN = salience network.

**Figure 3:**
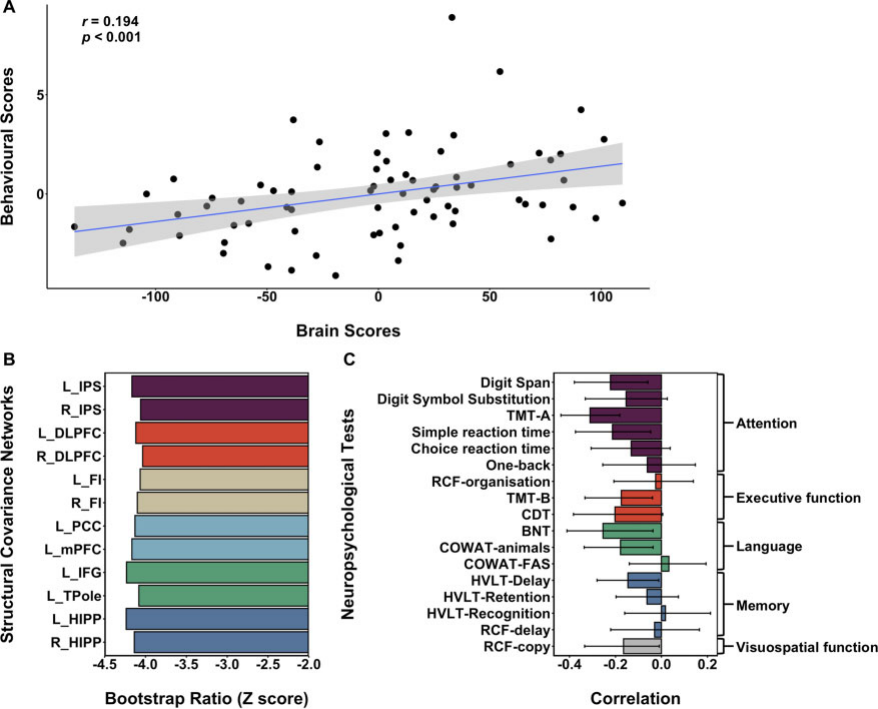
**Lower baseline integrity of SCNs was associated with greater impairment in cognitive performance in sub-acute stroke.** (**A**) A positive correlation between behavioural and brain scores suggested more damaged SCNs were associated with worse attention, executive function, language, memory and visuospatial function performance at 3-month post-stroke. (**B**) The bootstrap ratio (akin to a *Z*-score) demonstrated the contributions of each SCN to the covariance between SCNs and neuropsychological tests. (**C**) Seventeen neuropsychological tests showed extensive negative correlations with SCNs, particularly in the Digit Span Task, TMT-A and simple reaction time task within the attention domain, the TMT-B within the executive function domain, the BNT and COWAT-animals within the language domain, and the RCF-copy within the visuospatial domain. The error bars indicate 95% CI. Abbreviations: BNT = Boston Naming Test; CDT = clock-drawing test; COWAT = Controlled Oral Word Association Test; DAN = dorsal attention network; DMN = default mode network; ECN = executive control network; HVLT = Hopkins Verbal Learning Test; l_DLPFC = left dorsolateral prefrontal cortex; l_FI = left frontal insula; l_HIPP = left hippocampus; l_IFG = left inferior frontal gyrus; l_IPS = left intra-parietal sulcus; l_mPFC = left medial prefrontal cortex; l_PCC = left posterior cingulate cortex; l_TPole = left temporal pole; LN = language-related network; MN = memory network; r_DLPFC = right dorsolateral prefrontal cortex; r_HIPP = right hippocampus; r_FI = right frontal insula; r_IPS = right intra-parietal sulcus; RCF = Rey Complex Figure; SCN = structural covariance network; SN = salience network; TMT-A = trail-making test (**A**); TMT-B = trail-making test (**B**).

We derived canonical SCNs using 12 seeds from six major brain networks underlying cognitive function at the sub-acute timepoint (Fig. 2, surface rendered in Supplementary Materials Section 7, Supplementary Fig. 9). A LV1 significantly contributed to 12 SCNs (*P *=* *0.001) and explained 99.83% of the variance in the behavioural PLS model. There was a positive correlation between the behavioural and brain scores (*r *=* *0.194, *P *<* *0.001, Fig. 3A). All SCNs contributed fairly equally and significantly to LV1 with less than −2 bootstrap ratio (akin to *Z*-score) (Krishnan *et al.*, 2011) (Fig. 3B). The corresponding neuropsychological tests revealing the strongest correlations of LV1 were the Digit Span Task [*r* = −0.222; 95% confidence interval (CI) = −0.379 to −0.059], Trail-Making test (A) (TMT-A) (*r* = −0.311, 95% CI = −0.437 to −0.181) and Simple Reaction Time task (*r* = −0.213, 95% CI = −0.374 to −0.047) in the attention domain, the Trail-Making test (B) (TMT-B) (*r* = −0.175, 95% CI = −0.333 to −0.039) in the executive function domain, the Boston Naming Test (BNT) (*r* = −0.254, 95% CI = −0.411 to −0.037) and Controlled Oral Word Association Test (COWAT)-animals (*r* = −0.178, 95% CI = −0.337 to −0.037) in the language domain, the Hopkins Verbal Learning Test (HVLT)-Delay (*r* = −0.146, 95% CI = −0.280 to −0.012) in the memory domain and the Rey Complex Figure (RCF)-copy (*r* = −0.165, 95% CI = −0.334 to −0.009) in the visuospatial function domain (Fig. 3C). These results suggested that more damaged SCNs were related to worse attention, executive function, language, memory and visuospatial function performance at the sub-acute timepoint. An additional control analysis without correcting for infarct volume revealed highly similar results, indicating that our findings were robust (Supplementary Materials Section 4 and Supplementary Fig. 2). Further analyses controlling for global brain atrophy also replicated these findings (Supplementary Section 5.1 and Supplementary Fig. 4). Finally, we tested the model in an independent validation. Replicating the main results, a single LV explained 87.18% of the variance in the behavioural PLS model (*P *<* *0.001) and all 12 SCNs significantly contributed to this LV (Supplementary Materials Section 6.1 and Supplementary Fig. 6). The significant correlation between brain and behavioural scores was also replicated (*r *=* *0.325, *P *<* *0.001). Additional analyses controlling for GM/TIV or GM + WM/TIV again demonstrated similar findings (Supplementary Materials Section 6.1 and Supplementary Fig. 7).

### Relationship between changes in SCNs and changes in cognitive function

**Figure 4:**
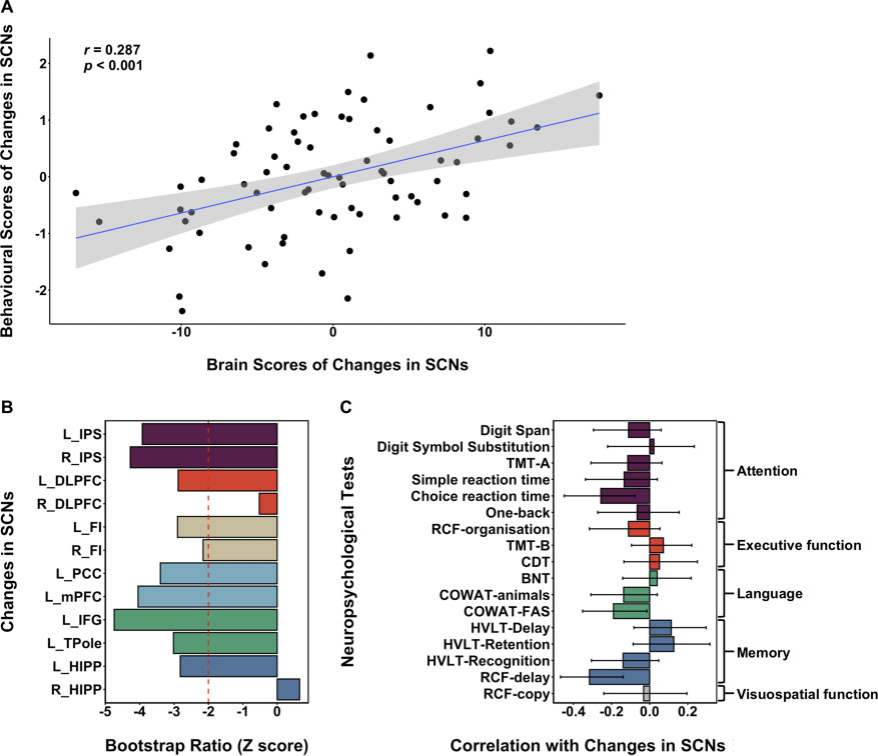
**Faster degradation of SCNs was associated with greater longitudinal decline in performance of attention, language and memory from 3-month to 1-year post-stroke.** (**A**) A positive correlation between changes in behavioural scores and changes in brain scores suggested that faster SCN degeneration was associated with greater longitudinal decline in neuropsychological tests from 3-month to 1-year post-stroke. (B) The bootstrap ratio demonstrated the contributions of each SCN to the covariance between SCNs and neuropsychological tests. (C) The significant correlation between each neuropsychological test and SCNs was shown in the choice reaction time task within the attention domain, the COWAT-FAS within the language domain and the RCF-delay within the memory domain. The error bars indicate 95% confidence interval. Abbreviations: BNT = Boston Naming Test; CDT = clock-drawing test; COWAT = Controlled Oral Word Association Test; DAN = dorsal attention network; DMN = default mode network; ECN = executive control network; HVLT = Hopkins Verbal Learning Test; l_DLPFC = left dorsolateral prefrontal cortex; l_FI = left frontal insula; l_HIPP = left hippocampus; l_IFG = left inferior frontal gyrus; l_IPS = left intra-parietal sulcus; l_mPFC = left medial prefrontal cortex; l_PCC = left posterior cingulate cortex; l_TPole = left temporal pole; LN = language-related network; MN = memory network; r_DLPFC = right dorsolateral prefrontal cortex; r_HIPP = right hippocampus; r_FI = right frontal insula; r_IPS = right intra-parietal sulcus; RCF = Rey Complex Figure; SCN = structural covariance network; SN = salience network; TMT-A = trail-making test (**A**); TMT-B = trail-making test (**B**).

We derived networks based on the covariance of the rate of change in GM volume from sub-acute to chronic stroke. Importantly, the regions showing significant co-variation with the seeds mirror those shown in the SCNs derived at the sub-acute timepoint (Fig. 2, surface rendered Supplementary Fig. 9). This confirms co-variation in the change in GM volume occurs within the domain-specific canonical networks, albeit to a reduced spatial extent. After applying behavioural PLS, one significant LV was identified for SCNs (*P *<* *0.001) and it explained 64.91% of variance of the PLS model. A positive correlation between the behavioural and the brain scores (*r *=* *0.287, *P *<* *0.001) was once again noted (Fig. 4A). All SCNs except right hippocampus (HIPP) and right dorsolateral prefrontal cortex (DLPFC) showed stable negative weightings (Fig. 4B). The corresponding neuropsychological tests showing significant correlations were the choice reaction time task (*r *=* *0.256, 95% CI = 0.449–0.078) in the attention domain, the controlled word association test-FAS in the language domain (*r *=* *0.188, 95% CI = 0.357–0.010) and RCF-delay (*r *=* *0.316, 95% CI = 0.468–0.139) in the memory domain (Fig. 4C). Highly similar results were found in a control analysis without correcting for infarct volume (Supplementary Materials Section 4 and Supplementary Fig. 3). In the split-half validation, we found a single LV (*P *<* *0.001) accounting for between 41.15 and 60.10% of the variance in the PLS model across 10 split-half samples (Supplementary Materials Section 6.2 and Supplementary Table 4). The first iteration of split-half validation analysis showed a correlation between behavioural change and change in SCN brain scores of similar magnitude to the discovery analysis (*r *=* *0.435, sample 1 and *r *=* *0.465, sample 2 (*P *<* *0.001), Supplementary Materials Section 6.2 and Supplementary Fig. 8).

## Discussion

Cognitive decline is common after ischaemic stroke (Hochstenbach *et al.*, 1998; Levine *et al.*, 2015) but has proved difficult to predict because the effects of stroke are not limited to the primary neuroanatomical location of brain damage. Functional networks based on correlated brain activity, and structural networks based on WM fibre connections have been extensively studied in stroke (Bullmore and Sporns, 2009; Grefkes and Fink, 2011, 2014) and reflect widespread disruption, despite focal and heterogenous damage caused by stroke. In contrast, there has been much less investigation of SCNs in stroke, despite their potential to clarify patterns of distributed atrophy, or reflect recovery-related plasticity (Spreng and Turner, 2013; Abela *et al.*, 2015). We used a novel, data-driven method, taking advantage of our unique longitudinal data to examine covariance in the rate of change in SCNs from sub-acute to chronic stroke. Crucially, we sought to determine if these SCNs and their longitudinal change had cognitive consequences by examining the relationship to cognitive performance and cognitive decline across domains. We show that cognitive decline after ischaemic stroke is associated with degeneration of canonical SCNs.

Structural covariance of the default mode, dorsal attention, executive control, salience, memory and language-related networks was associated with cognitive performance in the attention, executive function, language, memory and visuospatial domains, showing an association between topographical network organization in sub-acute stroke and cognitive performance. Structural covariance networks seeded from known network nodes replicated the topographical pattern of known functionally specific networks. Using a global ‘brain score’ and ‘behavioural score’ estimated from the PLS model, we found a significant correlation showing cognitive impairment associated with more damaged SCNs that we replicated in the independent validation data set. Structural covariance of major canonical networks is associated with cognitive performance in sub-acute stroke. Specifically, more damaged SCNs were related to deficits in attention, executive function, language, memory and visuospatial function. Attention was most implicated in this analysis with three tests of attention co-varying with the SCN LV, compared to a single test in other domains. This may reflect the frequency of attentional impairment seen in sub-acute stroke, or the overlap of attentional functions across other cognitive domains.

As a further test of whether SCN integrity was associated with cognitive impairment after stroke, we examined covariance in the rate of longitudinal change in major brain networks and related this to changes in cognitive performance between sub-acute and chronic stroke. Covariance networks mirrored the SCNs derived at the sub-acute timepoint, suggesting that GM volume changes from the sub-acute to chronic phase were occurring within established, domain-specific networks. The greatest changes, in terms of extent of distributed spatial co-varying patterns, were in the default mode network and dorsal attention network.

Degeneration of the major SCNs was associated with cognitive decline, specifically in the attention, memory and language domain. These results suggested that faster degradation of SCNs of bilateral dorsal attention, default mode, language-related networks as well as left dorsolateral prefrontal cortex, hippocampus and frontal insula was related to greater decline in attention, language and memory performance. These two findings are important, but should not be surprising, given that attentional deficits are a consistent feature of post-stroke cognitive impairment. Indeed, the most frequently impaired domains after ischaemic stroke are attention, memory and language (Gottesman and Hillis, 2010). Up to 70% of patients have impaired speed of processing and attention after stroke. (Hochstenbach *et al.*, 1998; Hyndman *et al.*, 2008; Barker-Collo *et al.*, 2009). Similarly, memory problems are a frequent complaint after stroke, with estimates around 23–55% of patients are affected at 3-month post-stroke and 11–31% affected at 1 year (Snaphaan and de Leeuw, 2007; das Nair *et al.*, 2016). Our study suggests at least some of this attention, memory and language impairment may be driven by widespread degeneration of SCNs from the sub-acute to chronic phase. Developmental SCN changes (Zielinski *et al.*, 2010), as well as in normal aging and neurological diseases (Evans, 2013; Spreng and Turner, 2013), have been well characterized. They have rarely associated with cognitive measures, and not been well investigated after ischaemic stroke.

What might be the mechanism resulting in widespread SCN changes, across all networks, after ischaemic stroke, even after accounting for age-related degeneration? Stroke may initiate or aggravate neurodegenerative processes above that seen in healthy aging (Cumming and Brodtmann, 2011). One plausible mechanism for widespread structural changes as the result of focal ischaemic stroke is secondary Wallerian degeneration due to disconnection between brain regions as a result of the stroke (Duering *et al.*, 2012). If a brain region, or multiple brain regions in the case of complex networks, is disconnected after stroke, there may be degeneration as a result of under-utilization of the disconnected region that results in volume loss (Duering *et al.*, 2012). Alternatively, stroke may initiate an ischaemic cascade that results in neurodegenerative processes, leading to widespread brain atrophy (Cumming and Brodtmann, 2011; Xing *et al.*, 2012; Shi *et al.*, 2019). Given the timescale of the atrophic changes (3-month and 1-year post-stroke) and how widespread they are in nature, a more plausible alternative is that stroke occurred on a background of accelerated atrophy as the result of cerebrovascular burden (Knopman and Hooshmand, 2017; Werden *et al.*, 2017). Future study should examine the clinical characteristics that predict widespread SCN degeneration associated with cognitive impairment.

The study should be interpreted in light of its limitations. As it is often the case, the heterogeneity observed within stroke cohorts precludes detailed examination of different profiles of cognitive impairments as the sample size of each sub-group was too small for adequate statistical power. We controlled for infarct volume in the analysis, but did not take into account location, again the heterogeneity of the stroke types and infarct locations, make sub-group analyses under-powered. We conducted an independent validation analysis and confirmed our main findings, namely one significant LV accounting for most of the variance in the seed PLS models. Fairly equal contributions from all SCNs as well as significant correlations between brain and behavioural scores were observed in the sub-acute model, which is similar to the findings in the discovery analysis. However, there were some differences related to the SCN profiles and neuropsychological tests that correlated with the LV in the longitudinal model. This may be the result of the sample size used in the validation sample (one-third of the discovery sample). Alternatively, this might reflect a degree of dynamic change in cognition at this timepoint. Cognition is likely to stabilize as the time from the stroke increases. Future study will examine the longitudinal effects at even longer, likely even more stable timepoints collected in this protocol (up to 5 years). As a group, the median stroke severity (as measured by mRS and NIHSS) was relatively mild. Although this may limit generalizability of the finding to cohorts with more severe stroke, it also raises the possibility that SCNs and cognition maybe even more disrupted when stroke is not as mild as in this cohort. Finally, we carefully chose the seeds to derive our SCNs based on the existing literature. Emerging large functional network atlases (Dosenbach *et al.*, 2010; Power et al., 2011; Yeo *et al.*, 2011) could be employed to facilitate seed definitions to produce SCNs (DuPre and Spreng, 2017). Future study should aim to replicate our findings to ensure it is robust to seed location.

## Summary

Cognitive decline after ischaemic stroke has been difficult to predict due to widespread effects of stroke on the brain. Using data-driven multivariate methods to examine cognition and canonical brain networks across cognitive domains, we show that structural covariance integrity of cognitive networks is associated with cognition at 3-month post-stroke and with longitudinal cognitive decline in attention, memory and language from sub-acute to chronic stroke. Structural covariance analyses of brain networks reveal widespread network disruptions associated with cognitive decline.

## Supplementary Material

fcaa155_Supplementary_Data

## Abbreviations

BNT =: Boston Naming Test;
CAT12 =: computational anatomy toolbox;
CDT =: clock-drawing test;
COWAT =: Controlled Oral Word Association Test;
CSF =: cerebrospinal fluid;
DAN =: dorsal attention network;
DMN =: default mode network;
ECN =: executive control network;
FLAIR =: fluid-attenuated inversion recovery;
GM =: grey matter;
HVLT =: Hopkins Verbal Learning Test;
l_DLPFC =: left dorsolateral prefrontal cortex;
l_FI =: left frontal insula;
l_HIPP =: left hippocampus;
l_IFG =: left inferior frontal gyrus;
l_IPS =: left intra-parietal sulcus;
l_mPFC =: left medial prefrontal cortex;
l_PCC =: left posterior cingulate cortex;
l_TPole =: left temporal pole;
LN =: language-related network;
LST =: lesion segmentation tool;
LV =: latent variable;
MDI =: missing data imputation;
MN =: memory network;
MOCA =: Montreal Cognitive Assessment;
MRI =: magnetic resonance imaging;
mRS =: modified Rankin Score;
NIHSS =: National Institute of Health Stroke Scale;
PLS =: partial least squares;
RCF =: Rey Complex Figure;
r_DLPFC =: right dorsolateral prefrontal cortex;
r_FI =: right frontal insula;
r_HIPP =: right hippocampus;
r_IPS =: right intra-parietal sulcus;
ROI =: region of interest;
SCN =: structural co-variance network;
SN =: salience network;
SPM =: Statistical Parametric Mapping;
TE =: echo time;
TMT-A =: trail-making test (A);
TMT-B =: trail-making test (B);
TR =: repetition time;
VBM =: voxel-based morphometry;
WM =: white matter
