# Supplementary material for: Degeneration of structural brain networks is associated with cognitive decline after ischaemic stroke
Source: Brain Commun. 2020 Sep 26;2(2):fcaa155. doi: 10.1093/braincomms/fcaa155 (PMC7751023; doi:10.1093/braincomms/fcaa155)
Supplement: fcaa155_Supplementary_Data [file fcaa155_supplementary_data.docx]

**Supplementary Materials**

**Table of Contents**

[Abbreviations 2](#_Toc50218919)

[1. Missing data percentage at subacute and chronic timepoints 3](#_Toc50218920)

[Supplementary Table 1. Domain-specific neuropsychological tests and percentage of missing data at subacute and chronic timepoint. 3](#_Toc50218921)

[2. Seed region and coordinates of structural covariance networks 4](#_Toc50218922)

[Supplementary Table 2. Selected seed region and coordinates of structural covariance networks. 4](#_Toc50218923)

[3. ROI seed and lesion overlap 5](#_Toc50218924)

[Supplementary Figure 1. Axial map showing distribution of lesions and overlap with seed ROIs. Seed regions shown in red. Colourbar shows number of patients with overlapping lesions. Neurological orientation (right depicted on the right). 5](#_Toc50218925)

[4. Infarct volume control analysis: Behavioural partial least squares analysis without controlling for infarct volume 6](#_Toc50218926)

[Supplementary Figure 2. Lower baseline integrity of structural covariance networks was associated with greater impairment in cognitive performance in subacute stroke. These relationships were not affected by infarct volume. 6](#_Toc50218927)

[Supplementary Figure 3. Faster degradation of structural covariance networks was associated with greater longitudinal decline in performance of attention, language and memory from 3-months to 1-year post-stroke (independent of infarct volume). 8](#_Toc50218928)

[5. Control for global atrophy: Discovery dataset 9](#_Toc50218929)

[5.1 Subacute timepoint 9](#_Toc50218930)

[Supplementary Figure 4. Lower baseline integrity of structural covariance networks was associated with greater impairment in cognitive performance in subacute stroke. These relationships were not driven by global atrophy. 10](#_Toc50218931)

[5.2 Chronic timepoint – controlling for longitudinal atrophy 12](#_Toc50218932)

[Supplementary Figure 5. Faster degradation of structural covariance networks was associated with greater longitudinal decline in performance of attention, language and memory from 3-months to 1-year post-stroke (independent of brain atrophy). 12](#_Toc50218933)

[6. Validation analysis 13](#_Toc50218934)

[Supplementary Table 3. Participant demographic and behavioural characteristics 14](#_Toc50218935)

[6.1 Validation analysis, independent dataset, subacute timepoint 15](#_Toc50218936)

[Supplementary Figure 6. Lower baseline integrity of structural covariance networks was associated with greater impairment in attention in validation dataset 15](#_Toc50218937)

[Supplementary Figure 7. Lower baseline integrity of structural covariance networks was associated with greater impairment in cognitive performance in validation dataset. These relationships were not driven by global atrophy. 16](#_Toc50218938)

[6.2 Validation analysis, split half analysis, longitudinal changes 17](#_Toc50218939)

[Supplementary Table 4. Split half validation analysis, longitudinal changes. 17](#_Toc50218940)

[Supplementary Figure 8. Faster degradation of structural covariance networks was associated with greater longitudinal cognitive decline from 3-months to 1-year post-stroke in split half validation dataset. 18](#_Toc50218941)

[6.3 Validation analysis, split half analysis, longitudinal changes – global atrophy controlled 19](#_Toc50218942)

[Supplementary Table 5. Split half validation analysis, longitudinal changes – global atrophy controlled. 19](#_Toc50218943)

[7. Surface-rendered structural covariance networks in subacute and chronic patients 20](#_Toc50218944)

[Supplementary Figure 9. Surface-rendered structural covariance networks in subacute and chronic stroke patients. 22](#_Toc50218945)

# **Abbreviations**

### BNT – Boston Naming Test

### CDT – clock-drawing test

### COWAT – Controlled Oral Word Association Test

### DAN – dorsal attention network

### DMN – default mode network

### ECN – executive control network

### HVLT – Hopkins Verbal Learning Test

### l_DLPFC – left dorsolateral prefrontal cortex

### l_FI – left frontal insula

### l_HIPP – left hippocampus

### l_IFG – left inferior frontal gyrus

### l_IPS – left intraparietal sulcus

### l_mPFC – left medial prefrontal cortex

### l_PCC – left posterior cingulate cortex

### l_TPole – left temporal pole

### LN – language-related network

### MN – memory network

MoCA – Montreal Cognitive Assessment

mRS – modified Rankin Score

NIHSS – National Institute of Health Stroke Scale

### r_DLPFC – right dorsolateral prefrontal cortex

### r_HIPP – right hippocampus

### r_FI – right frontal insula

### r_IPS – right intraparietal sulcus

### RCF – Rey Complex Figure

### SCN – structural covariance network

### SN – salience network

### TMT-A – trail-making test (A)

### TMT-B – trail-making test (B).

# **1. Missing data percentage at subacute and chronic timepoints**

## Supplementary Table 1. Domain-specific neuropsychological tests and percentage of missing data at subacute and chronic timepoint.

| Cognitive Domain | Neuropsychological Test | Missing Data | |
| --- | --- | --- | --- |
|  |  | **Subacute** | **Chronic** |
| Attention | Digit Span Task (WAIS – Third Edition) | 3% | 3% |
|  | Digit Symbol Substitution Task (WAIS – Third Edition) | 3% | 3% |
|  | TMT-A | 1% | 1% |
|  | Simple reaction time task | 1% | 1% |
|  | Choice reaction time task | 1% | 1% |
|  | One-back task | 1% | 3% |
| Executive Function | RCF-organisation | 4% | 5% |
|  | TMT-B | 3% | 3% |
|  | CDT | 1% | 1% |
| Language | COWAT-animals | 5% | 5% |
|  | COWAT-FAS | 4% | 4% |
|  | BNT | 3% | 3% |
| Memory | HVLT-Delay | 0% | 1% |
|  | HVLT-Retention | 0% | 1% |
|  | HVLT-Recognition | 0% | 1% |
|  | RCF-delay | 3% | 3% |
| Visuospatial Function | RCF-copy | 3% | 4% |

Abbreviations: BNT – Boston Naming Test; CDT – Clock-Drawing Test; COWAT – Controlled Oral Word Association Test; HVLT – Hopkins Verbal Learning Test; RCF – Rey Complex Figure; TMT-A – Trail-making test (A); TMT-B – Trail-Making Test (B); WAIS – Weschler Adult Intelligence Scale.

# **2. Seed region and coordinates of structural covariance networks**

## Supplementary Table 2. Selected seed region and coordinates of structural covariance networks.

|  |  | Coordinates | | |  |
| --- | --- | --- | --- | --- | --- |
| Network | **Seed Region** | **X** | **Y** | **Z** | **Reference** |
| Dorsal Attention Network | Left IPS | -31 | -55 | 54 | (Fox *et al.*, 2006) |
|  | Right IPS | 31 | -55 | 54 | (Fox *et al.*, 2006) |
| Executive Control Network | Left DLPFC | -44 | 36 | 20 | (Seeley *et al.*, 2007) |
|  | Right DLPFC | 44 | 36 | 20 | (Seeley *et al.*, 2007) |
| Salience Network | Left FI | -38 | 26 | -10 | (Seeley *et al.*, 2007) |
|  | Right FI | 38 | 26 | -10 | (Seeley *et al.*, 2007) |
| Default mode network | Left PCC | -7 | -43 | 33 | (Sridharan *et al.*, 2008; Chong *et al.*, 2017) |
|  | Left mPFC | -16 | 48 | 44 | (Greicius *et al.*, 2003; Vipin *et al.*, 2018) |
| Language-related Network | Left TPole | -38 | 10 | -28 | (Zielinski *et al.*, 2010) |
|  | Left IFG | -50 | 18 | 7 | (Zielinski *et al.*, 2010) |
| Memory Network | Left HIPP | -21 | -25 | -14 | (Koechlin *et al.*, 1999; Vincent *et al.*, 2008) |
|  | Right HIPP | 24 | -19 | -21 | (Koechlin *et al.*, 1999; Vincent *et al.*, 2008) |

Coordinates shown are in Montreal Neurological Institute normalized space. Abbreviations: DLPFC – dorsolateral prefrontal cortex; FI – Frontal Insula; HIPP – hippocampus; IFG – Inferior frontal gyrus; IPS – intraparietal sulcus; MNI – Montreal Neurological Institute; mPFC – medial prefrontal cortex; PCC – posterior cingulate cortex; TPole – temporal pole.

# **3. ROI seed and lesion overlap**

We used FSLMATHS to calculate the percentage of voxels overlapping between the ROI seeds and the lesion maps, both transformed into MNI space. We calculated that the percentage of voxels overlapping between the seeds and infarcts in the group map totalled less than 0.06%. It is therefore unlikely that damage to the seed regions cause by the infarcts is driving the structural covariance networks observed.

## Supplementary Figure 1. Axial map showing distribution of lesions and overlap with seed ROIs. Seed regions shown in red. Colourbar shows number of patients with overlapping lesions. Neurological orientation (right depicted on the right).

# **4. Infarct volume control analysis: Behavioural partial least squares analysis without controlling for infarct volume**

To examine the influence of infarct volume in confounding the behavioural PLS analysis, we performed two separate analyses without controlling for the log-transformed infarct volume, following the methods outlined for the discovery dataset analysis. The unstandardised residual brain scores (i.e., SCN scores) were input to the following behavioural PLS analyses.

The results of the infarct volume control analysis are illustrated in Supplementary Figure 2 and S3.


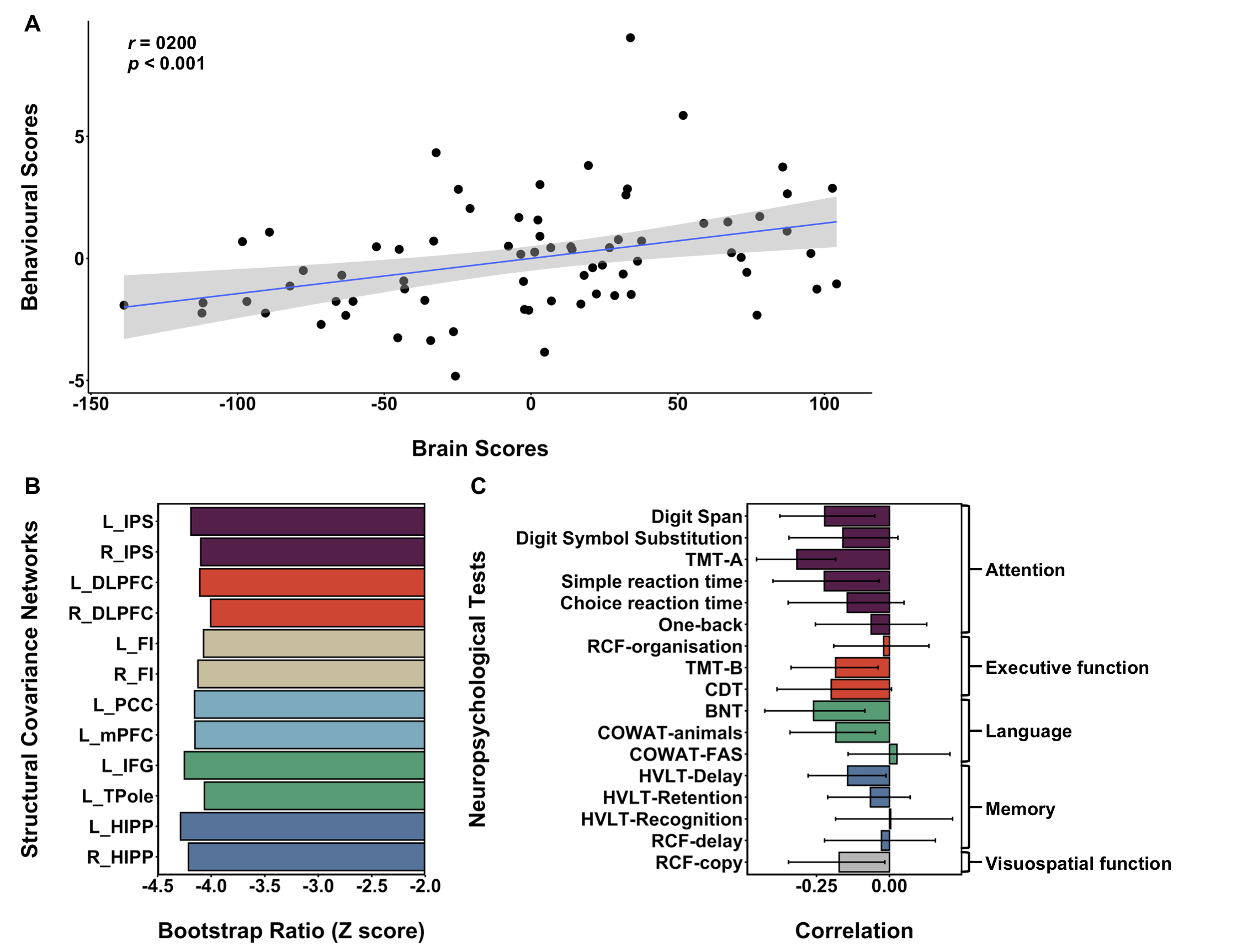


## Supplementary Figure 2. Lower baseline integrity of structural covariance networks was associated with greater impairment in cognitive performance in subacute stroke. These relationships were not affected by infarct volume.

### (A) A positive correlation between behavioural and brain scores suggested more damaged SCNs were associated with worse attention, executive function, language, memory, and visuospatial function performance at 3-months post-stroke. (B) The contributions of each SCN to the covariance between SCNs and neuropsychological tests were revealed by the bootstrap ratio. (C) Extensive negative correlations between SCNs and 17 neuropsychological tests were shown, particularly in the Digit Span Task (r = -0.222, 95% C.I. -0.376 – -0.051), TMT-A (r = -0.317, 95% C.I. -0.456 – -0.185), and simple reaction time task (r = -0.223, 95% C.I. -0.399 – -0.035) within the attention domain, the TMT-B (r = -0.185, 95% C.I. -0.337 – -0.039) and CDT (r = -0.200, 95% C.I. -0.385 – -0.007) within the executive function domain, the BNT (r = -0.260, 95% C.I. -0.427 – -0.084) and COWAT-animals (r = -0.184, 95% C.I. -0.341 – -0.048) within the language domain, the HVLT-Delay (r = -0.143, 95% C.I. -0.279 – -0.012) within the memory domain, and the RCF-copy (r = -0.172, 95% C.I. -0.346 – -0.016) within the visuospatial domain. This infarct volume control analysis also revealed a significant latent variable which could explain 99.84% of the variance of the PLS model.

### The error bars indicate 95% confidence interval.


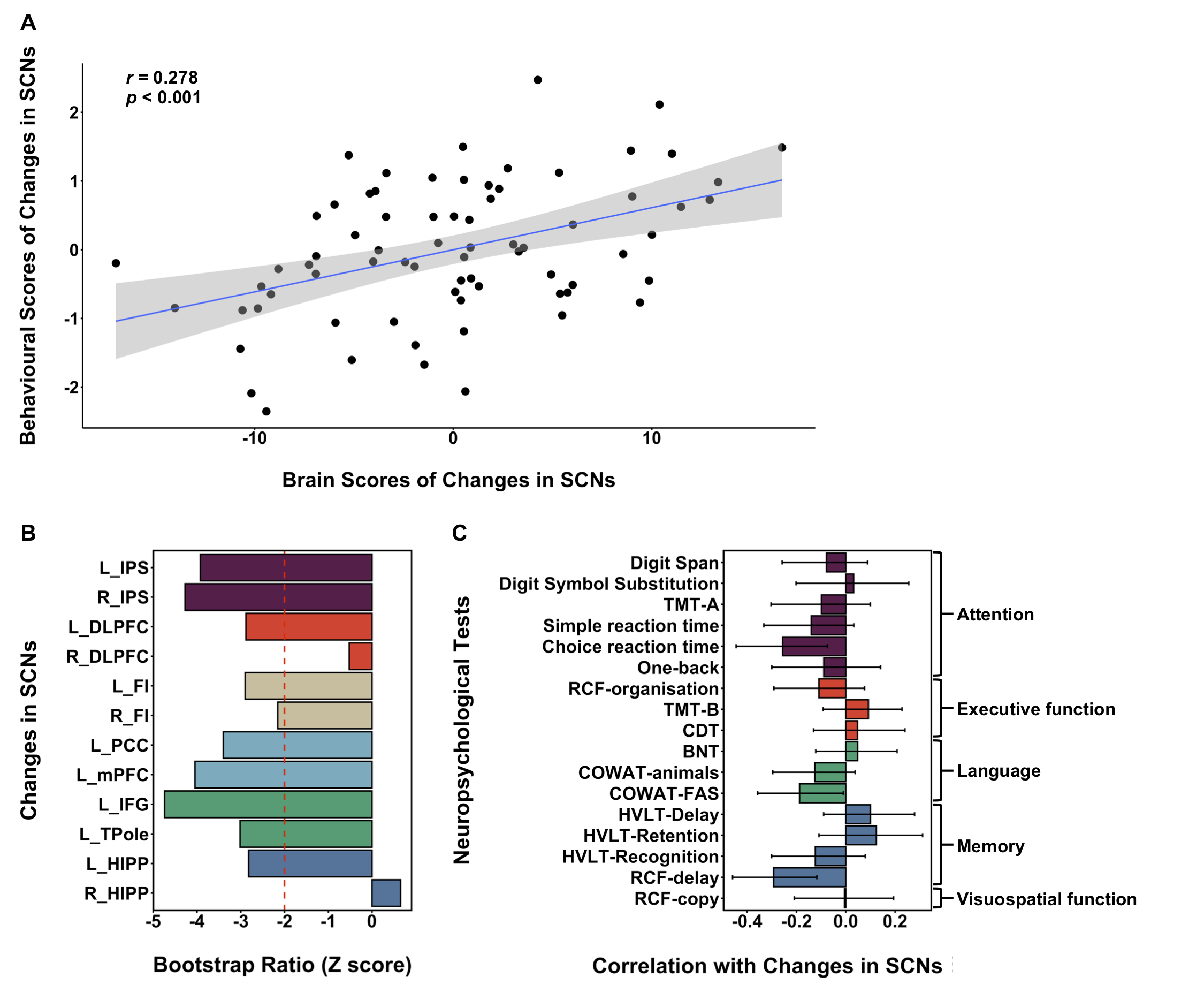


## Supplementary Figure 3. Faster degradation of structural covariance networks was associated with greater longitudinal decline in performance of attention, language and memory from 3-months to 1-year post-stroke (independent of infarct volume).

### (A) A positive correlation between behavioural and brain scores suggested faster SCN decline was associated with greater longitudinal decline in performance in neuropsychological tests from 3-months to 1-year post-stroke. (B) The contribution of each SCN to the covariance between SCNs and neuropsychological tests were revealed by the bootstrap ratio. (C) The significant correlation between each neuropsychological test and SCNs was shown in the choice reaction time task (r = -0.256, 95% C.I. -0.445 – -0.074) within the attention domain, the COWAT-FAS (r = -0.188, 95% C.I. -0.357 – -0.010) within the language domain, and the RCF-delay (r = -0.293, 95% C.I. -0.459 – -0.117) within the memory domain. A significant latent variable was found in the infarct volume control analysis and it could explain 63.72% of the variance of the PLS model. The error bars indicate 95% confidence interval.

# **5. Control for global atrophy: Discovery dataset**

To ensure our results were not driven by global atrophy, we replaced total intracranial volume as a confounding variable with global atrophy estimated as 1. grey matter volume normalised by total intracranial volume (GM/TIV) and 2. total brain volume (grey and white matter volume) as a proportion of total intracranial volume (GM+WM/TIV).

# **5.1 Subacute timepoint**

We noted no changes to the overall pattern of significant results when controlling for grey matter/total intracranial volume (Supplementary Figure 4.1) or grey and white matter/total intracranial volume (Supplementary Figure 4.2). In both analysis, one significant latent variable accounted for 99.78% and 99.68% of the variance in the behavioural PLS model, respectively. The significant correlation between behavioural and brain scores remained in both control analyses. We are therefore confident the results are not driven by global atrophy as estimated in these two different ways.


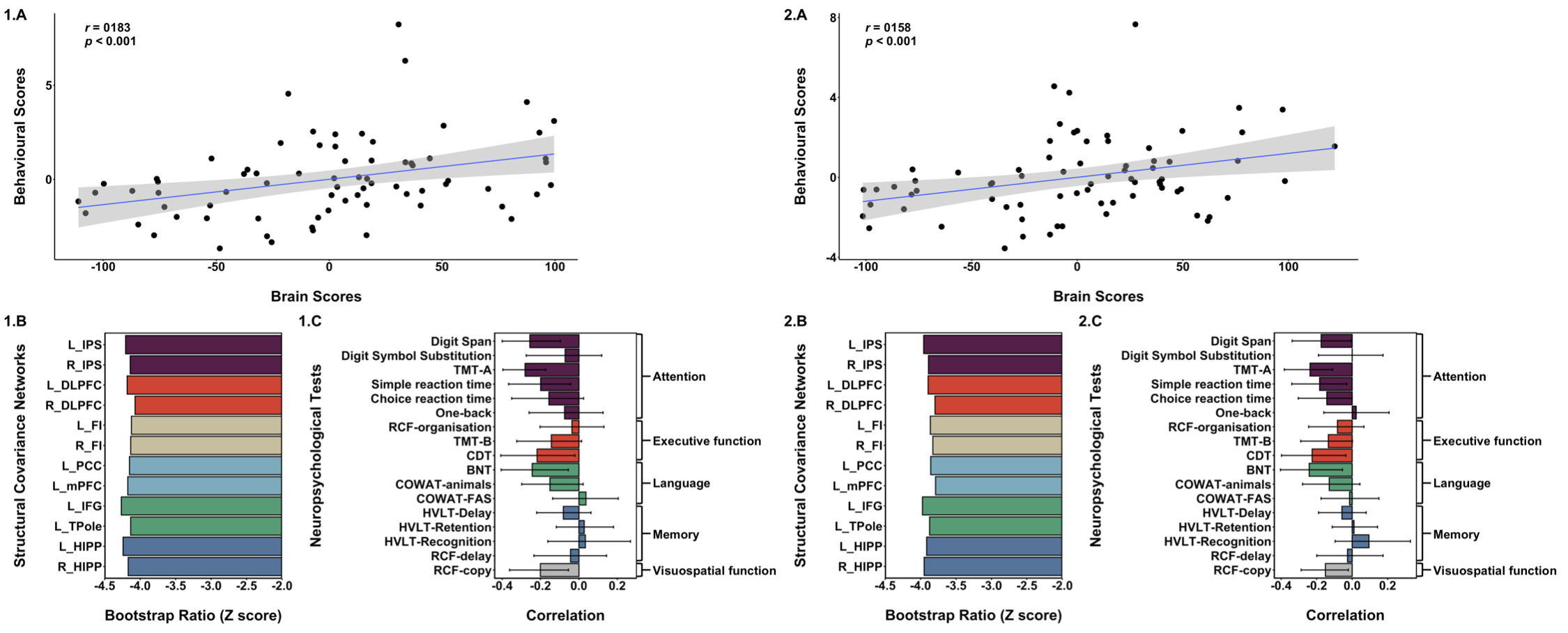


## Supplementary Figure 4. Lower baseline integrity of structural covariance networks was associated with greater impairment in cognitive performance in subacute stroke. These relationships were not driven by global atrophy.

### Panel 1 represents results after controlling for grey matter/total intracranial volume and panel 2 represents results after controlling for grey and white matter/total intracranial volume. (1A) A positive correlation between behavioural and brain scores suggested more damaged SCNs were associated with worse attention, executive function, language, and visuospatial function performance at 3-months post-stroke. (1B) The contributions of each SCN to the covariance between SCNs and neuropsychological tests were revealed by the bootstrap ratio, which indicated a fairly equal contribution of SCNs. (1C) Extensive negative correlations between SCNs and 17 neuropsychological tests were shown, particularly in the Digit Span Task (r = -0.255, 95% C.I. -0.400 – -0.096), TMT-A (r = -0.366, 95% C.I. -0.517 – -0.213), and simple reaction time task (r = -0.199, 95% C.I. -0.367 – -0.044) within the attention domain, the CDT (r = -0.318, 95% C.I. -0.407 – -0.021) within the executive function domain, the BNT (r = -0.243, 95% C.I. -0.406 – -0.055) within the language domain, and the RCF-copy (r = -0.201, 95% C.I. -0.362 – -0.054) within the visuospatial domain. (2A) Similar to 1A, a positive correlation was also noted in the analysis controlling for grey and white matter/total intracranial volume. (2B) Similar to 1B, each SCN contributed comparatively. (2C) Similar to 1C, significant correlations between SCNs and neuropsychological tests were in the Digit Span Task (r = -0.175, 95% C.I. -0.338 – -0.008), TMT-A (r = -0.238, 95% C.I. -0.382 – -0.110), and simple reaction time task (r = -0.183, 95% C.I. -0.340 – -0.031) within the attention domain, the CDT (r = -0.226, 95% C.I. -0.398 – -0.035) within the executive function domain, the BNT (r = -0.242, 95% C.I. -0.404 – -0.054) within the language domain, and the RCF-copy (r = -0.151, 95% C.I. -0.288 – -0.022) within the visuospatial domain.

The error bars indicate 95% confidence interval.

# **5.2 Chronic timepoint – controlling for longitudinal atrophy**

For the longitudinal analysis we additionally controlled for scan interval (time between subacute and chronic MRI scan) and longitudinal atrophy estimated as the difference between the atrophy measure at the chronic timepoint and the subacute timepoint. As with the subacute timepoint, we did this for GM/TIV and GM+WM/TIV. Here, the first latent variable accounted for 39.43% (GM/TIV) and 54.02% (GM+WM/TIV) of the variance in the PLS model. The main results were largely unchanged including significant correlations of similar magnitude between behavioural change and change in brain scores (Supplementary Figure 5).


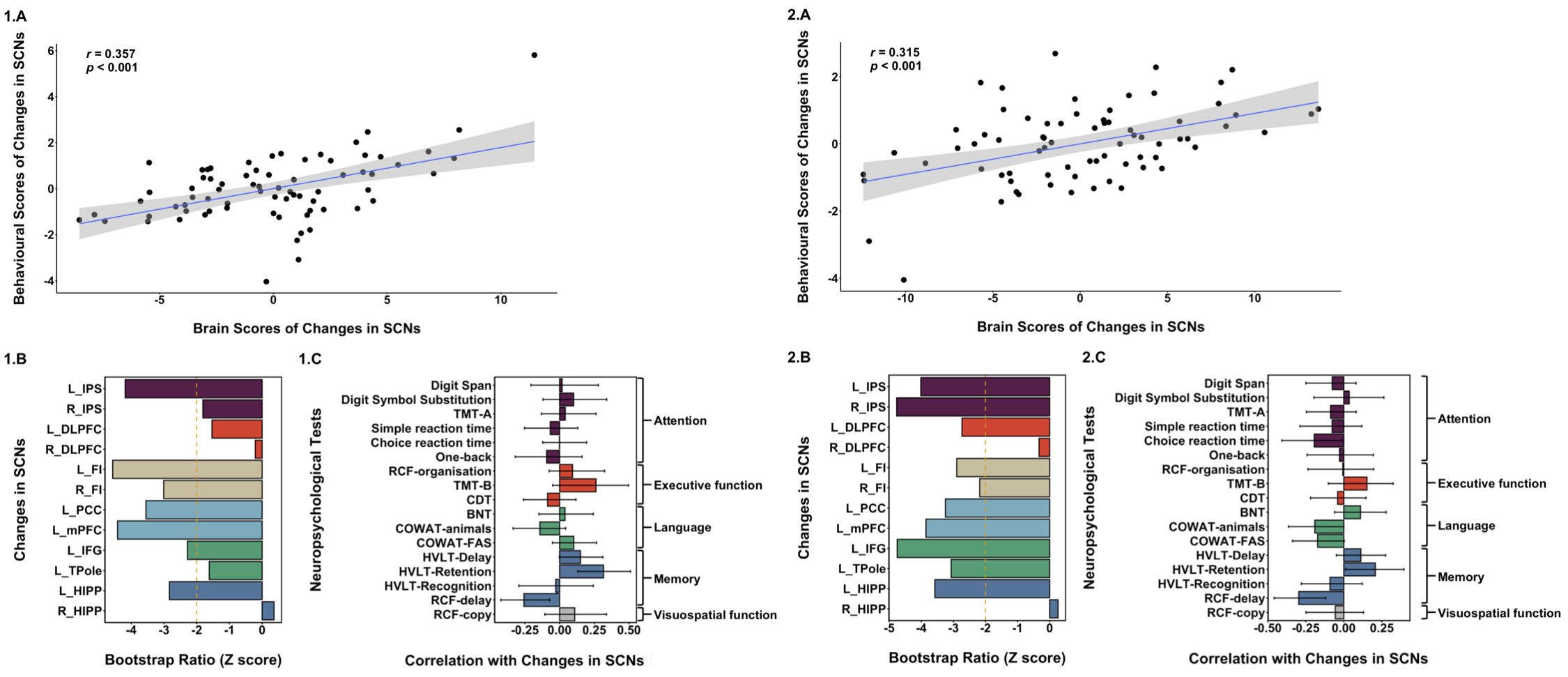


## Supplementary Figure 5. Faster degradation of structural covariance networks was associated with greater longitudinal decline in performance of attention, language and memory from 3-months to 1-year post-stroke (independent of brain atrophy).

Panel 1 represents results after controlling for grey matter/total intracranial volume and panel 2 represents results after controlling for grey and white matter/total intracranial volume. (1A, 2A) A positive correlation between behavioural and brain scores suggested faster SCN decline was associated with greater longitudinal decline in performance in neuropsychological tests from 3-months to 1-year post-stroke. (1B, 2B) The contribution of each SCN to the covariance between SCNs and neuropsychological tests were revealed by the bootstrap ratio. (1C) The significant correlations between each neuropsychological test and SCNs were shown in the HVLT-Retention (r = 0.317, 95% C.I. 0.130 – 0.511) and RCF-delay (r = -0.256, 95% C.I. -0.424 – -0.071) within the memory domain. (2C) The significant correlations between each neuropsychological test and SCNs were noted in the Choice reaction time task (r = -0.194, 95% C.I. -0.407– 0.009) within the attention domain and RCF-delay (r = -0.296, 95% C.I. -0.457 – -0.119) within the memory domain.

# **6. Validation analysis**

To investigate the reproducibility of our findings, we performed validation analyses using an independent sample with 26 participants recruited from the same cohort of the original sample (Brodtmann *et al.*, 2014). After identical quality control to the discovery dataset, data from four patients was excluded due to movement or lack of cognitive data. To ensure the two samples were comparable, independent *t*-tests were executed for continuous variables including age, education, infarct volume, Montreal Cognitive Assessment (MoCA), and scan interval. Chi-square tests were used for categorical variables including sex and handedness. Median tests were performed for ordinal variables including National Institute of Health Stroke Scale (NIHSS) and modified Rankin Score (mRS) at both admission and three months. No significant differences between the original and independent samples were noted (Supplementary Table 3). Identical procedures of SCN analysis and validation analysis of behavioural PLS were followed. A latent variable was found and it explained 87.18% of the variance in the PLS model (Supplementary Figure 6).

We additionally controlled for GM/TIV or GM+WM/TIV in the validation analysis. Again, we replicated the main pattern of results, finding one significant latent variable accounting for 98.11% and 98.20% of the variance in the PLS model respectively. In both control analyses, all SCNs contributed fairly equally to the latent variable (less than -2 bootstrap ratio across all SCNs). The correlation between behavioural and brain scores remained, again at similar magnitude, *r* = 0.330, *p* < 0.001 (GM/TIV) and *r* = 0.314, *p* < 0.001 (GM+WM/TIV). We are confident that the validation is not driven by these estimated of global atrophy either. The results of the control analyses are illustrated in Supplementary Figure 7.

## Supplementary Table 3. Participant demographic and behavioural characteristics

| Demographics | Discovery sample | Validation sample | *p*-value  (2-tailed) |
| --- | --- | --- | --- |
| Age (years), mean (SD) | 67.41 (12.13) | 68.77 (9.65) | 0.631 |
| Sex (male/female) | 51/22 | 16/6 | 0.796 |
| Handedness (left/right) | 6/67 | 1/21 | 0.563 |
| Education (years), mean (SD) | 12.89 (3.75) | 12.36 (4.03) | 0.572 |
| Infarct volume (mm^3^), mean (SD) | 5786.62 (9316.65) | 12377.65 (18062.69) | 0.112 |
| NIHSS on admission, median (25^th^, 75^th^ percentile) | 2 (1,4) | 2 (1,3) | 0.893 |
| NIHSS at three months, median (25^th^, 75^th^ percentile) | 0 (0,2) | 1 (0,1.25) | 0.289 |
| mRS on admission, median (25^th^, 75^th^ percentile) | 1 (1,2) | 1 (1,2) | 0.683 |
| mRS at three months, median (25^th^, 75^th^ percentile) | 1 (1,2) | 1 (0.75,2) | 0.120 |
| MoCA, mean (SD) | 24.26 (3.43) | 24.00 (2.62) | 0.869 |
| Scan interval (days), mean (SD) | 276.92 (26.14) | 281.95 (30.34) | 0.447 |

Abbreviations: MoCA – Montreal Cognitive Assessment; mRS – modified Rankin Score; NIHSS – National Institute of Health Stroke Scale; SD – standard deviation.

# **6.1 Validation analysis, independent dataset, subacute timepoint**


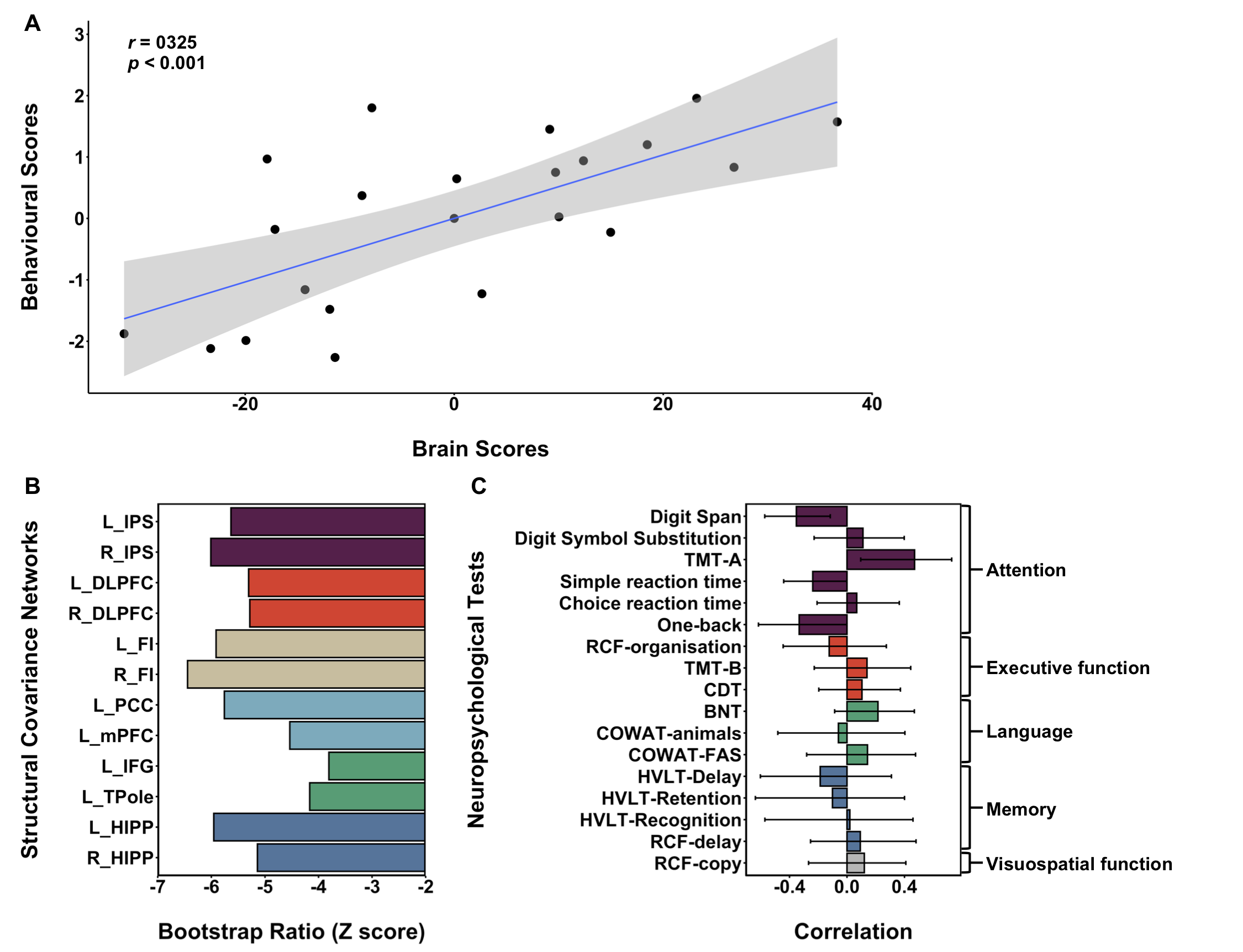


## Supplementary Figure 6. Lower baseline integrity of structural covariance networks was associated with greater impairment in attention in validation dataset

### This validation analysis demonstrated a significant latent variable which could explain 87.18% of the variance of the PLS model. (A) A positive correlation between behavioural and brain scores suggested more damaged SCNs were associated with worse attention, executive function, language, and visuospatial function performance at 3-months post-stroke. (B) The contributions of each SCN to the covariance between SCNs and neuropsychological tests were revealed by the bootstrap ratio. (C) Significant correlations between SCNs and 17 neuropsychological TMT-A (r = 0.470, 95% C.I. 0.095 – 0.728) within the attention domain.

### The error bars indicate 95% confidence interval.


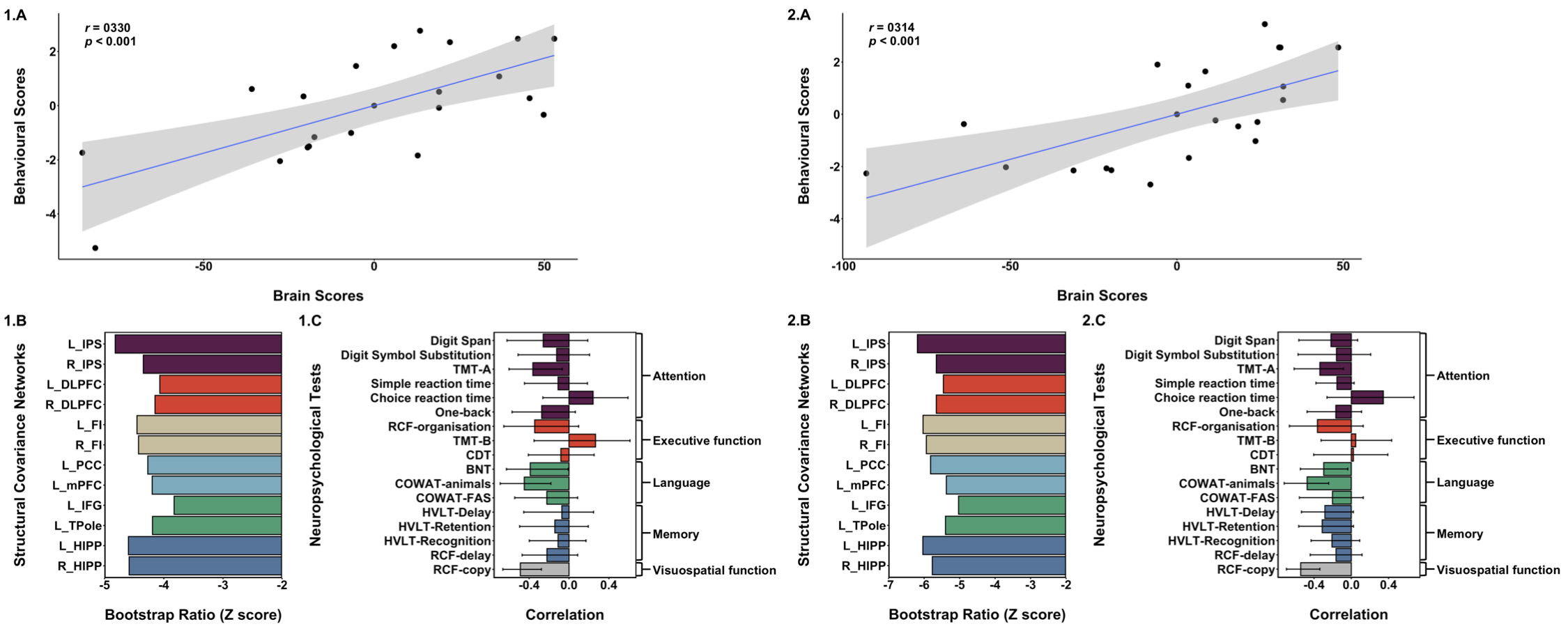


## Supplementary Figure 7. Lower baseline integrity of structural covariance networks was associated with greater impairment in cognitive performance in validation dataset. These relationships were not driven by global atrophy.

### Panel 1 represents results after controlling for grey matter/total intracranial volume and panel 2 represent results after controlling for grey and white matter/total intracranial volume. (1A, 2A) A positive correlation between behavioural and brain scores suggested more damaged SCNs were associated with worse attention, executive function, language, and visuospatial function performance at 3-months post-stroke. (1B, 2B) The contributions of each SCN to the covariance between SCNs and neuropsychological tests were revealed by the bootstrap ratio, which indicated a fairly equal contribution of SCNs. (1C) Extensive negative correlations between SCNs and 17 neuropsychological tests were shown in the TMT-A (r = -0.369, 95% C.I. -0.604 – -0.066) within the attention domain, the COWAT-animals (r = -0.449, 95% C.I. -0.693 – -0.181) within the language domain, and the RCF-copy (r = -0.489, 95% C.I. -0.666 – -0.277) within the visuospatial domain. (2C) Similar to 1C, significant correlations between SCNs and neuropsychological tests were in the TMT-A (r = -0.337, 95% C.I. -0.616 – -0.084) within the attention domain, the BNT (r = -0.296, 95% C.I. -0.547 – -0.038) and COWAT-animals (r = -0.474, 95% C.I. -0.422 – -0.242) within the language domain, and the RCF-copy (r = -0.543, 95% C.I. -0.698 – -0.337) within the visuospatial domain.

### The error bars indicate 95% confidence interval.

# **6.2 Validation analysis, split half analysis, longitudinal changes**

Due to a lack of data of sufficient quality at the chronic timepoint (total n = 14), we were unable to do a validation on an independent dataset for the chronic timepoint. We ran a split half analysis, randomly selecting patients to groups (n = 36 and 37, respectively) and repeating this five times to generate ten samples following the procedure and corrections outlined for the longitudinal discovery dataset. Supplementary Figure 8 shows the results of one iteration of the split half analysis. The first latent variable accounted for 45.40% of the variance in the PLS model in sample 1 and 60.10% of the variance in sample 2. The pattern of results were similar across validation samples, and a significant correlation between behavioural and brain score change was maintained at *r* = 0.438, *p* < 0.001 in sample 1 and *r* = 0.465 *p* < 0.001 in sample 2.

## Supplementary Table 4. Split half validation analysis, longitudinal changes.

| Iteration | Latent variable variance explained | | p-value | |
| --- | --- | --- | --- | --- |
|  | **Sample 1** | **Sample 2** | **Sample 1** | **Sample 2** |
| 1 | 45.40% | 60.10% | < 0.001 | < 0.001 |
| 2 | 50.90% | 52.98% | < 0.001 | < 0.001 |
| 3 | 53.38% | 53.96% | < 0.001 | < 0.001 |
| 4 | 43.36% | 56.55% | < 0.001 | < 0.001 |
| 5 | 59.70% | 41.15% | < 0.001 | < 0.001 |


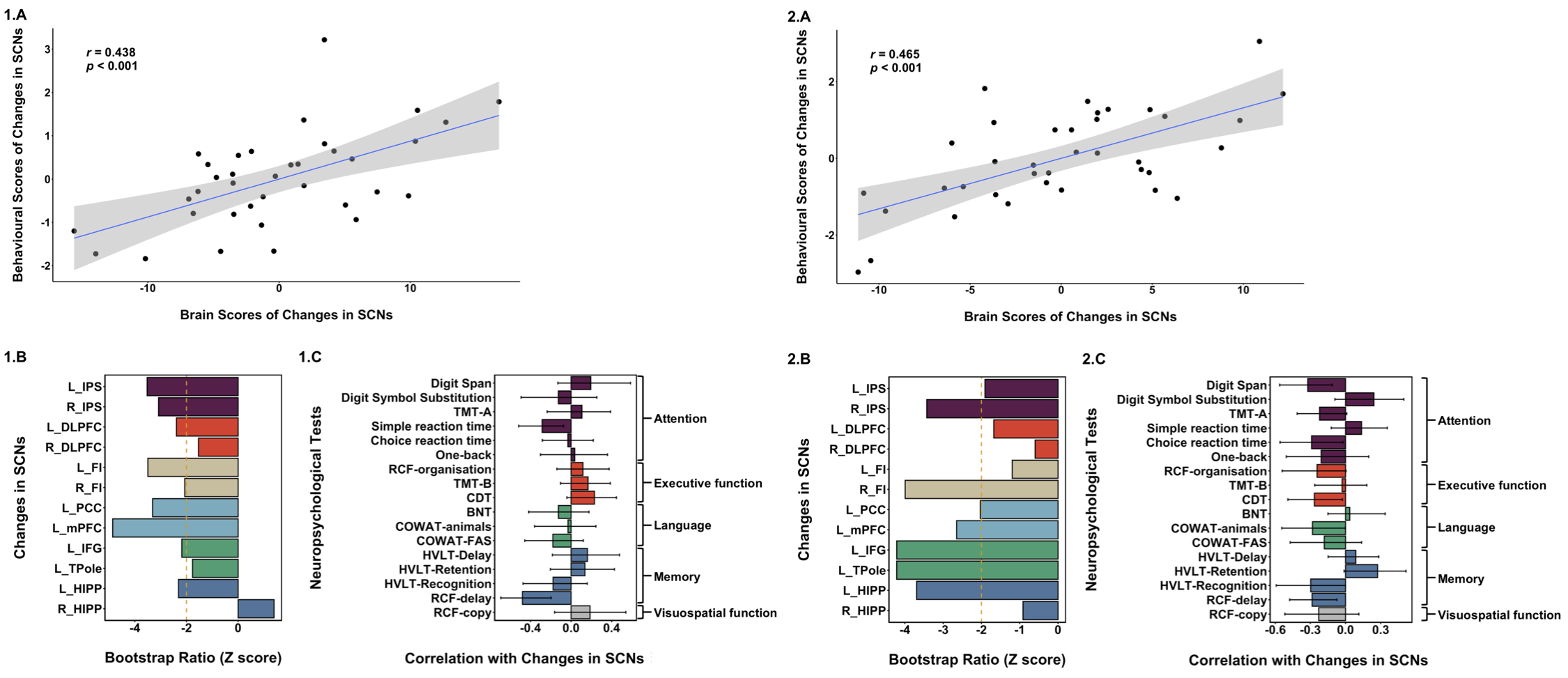


## Supplementary Figure 8. Faster degradation of structural covariance networks was associated with greater longitudinal cognitive decline from 3-months to 1-year post-stroke in split half validation dataset.

Panel 1 and 2 represent behavioural PLS results from two non-overlapping samples of one representative split-half run. The remaining 4 runs demonstrated similar results. (1A, 2A) A positive correlation between behavioural and brain scores suggested faster SCN decline was associated with greater longitudinal decline in performance in neuropsychological tests from 3-months to 1-year post-stroke. (1B, 2B) The contribution of each SCN to the covariance between SCNs and neuropsychological tests were revealed by the bootstrap ratio. (1C) The significant correlations between each neuropsychological test and SCNs were shown in the Simple reaction time task (r = -0.284, 95% C.I. 0.516 – 0.074) within the attention domain and RCF-delay (r = -0.479, 95% C.I. -0.694 – -0.196) within the memory domain. (2C) The significant correlations between each neuropsychological test and SCNs were noted in the Digit Span (r = -0.322, 95% C.I. -0.562– 0.112) and Choice reaction time task (r = -0.289, 95% C.I. -0.559– 0.022) within the attention domain, CDT (r = -0.265, 95% C.I. -0.493– 0.027) within the executive function domain, COWAT-animals (r = -0.282, 95% C.I. -0.544– 0.002) within the language domain, and RCF-delay (r = -0.284, 95% C.I. -0.477 – -0.073) within the memory domain.

# **6.3 Validation analysis, split half analysis, longitudinal changes – global atrophy controlled**

We ran the split half analysis additionally controlling for global atrophy as estimated by GM+WM/TIV. We once again found broadly similar results to the longitudinal discovery dataset and present the results of the 10 split half samples in Supplementary Table 5.

## Supplementary Table 5. Split half validation analysis, longitudinal changes – global atrophy controlled.

| Iteration | Latent variable variance explained | | p-value | |
| --- | --- | --- | --- | --- |
|  | **Sample 1** | **Sample 2** | **Sample 1** | **Sample 2** |
| 1 | 50.66% | 54.80% | < 0.001 | < 0.001 |
| 2 | 48.33% | 58.05% | < 0.001 | < 0.001 |
| 3 | 53.81% | 55.18% | < 0.001 | < 0.001 |
| 4 | 42.18% | 52.56% | < 0.001 | = 0.001 |
| 5 | 56.07% | 41.87% | < 0.001 | < 0.001 |

# **7. Surface-rendered structural covariance networks in subacute and chronic patients**

To enable visualisation of the entire networks at the subacute and longitudinal timepoints we surface rendered all the SCNs.


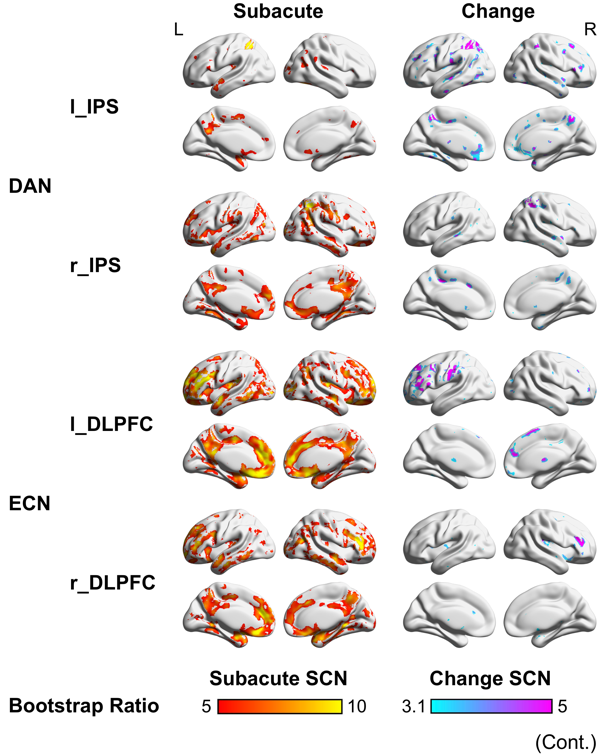


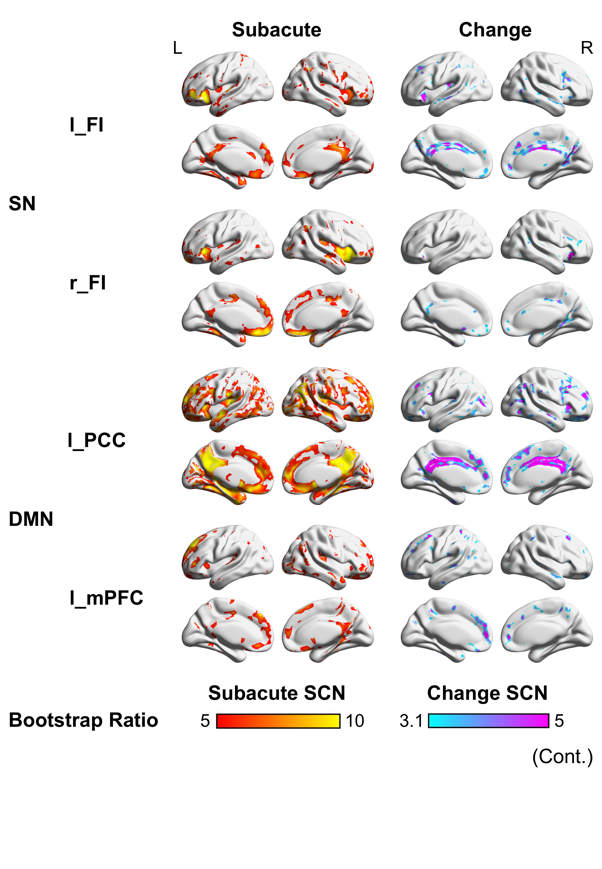


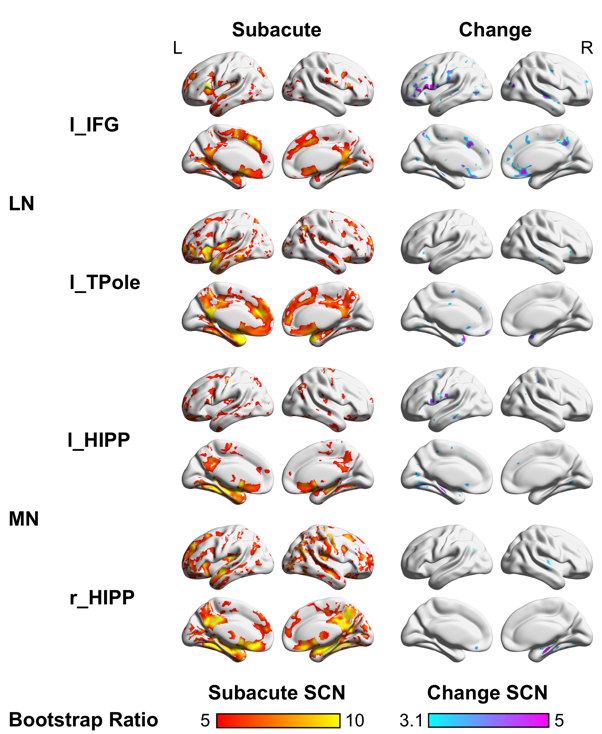


## Supplementary Figure 9. Surface-rendered structural covariance networks in subacute and chronic stroke patients.

The derived structural covariance networks using twelve seeds from six canonical brain networks at the subacute timepoint were depicted in the middle column in hot colour and those for change from subacute to chronic stroke were depicted in the right column in cold colour.

**REFERENCES**

Brodtmann A, Werden E, Pardoe H, Li Q, Jackson G, Donnan G*, et al.* Charting cognitive and volumetric trajectories after stroke: Protocol for the cognition and neocortical volume after stroke (CANVAS) study. International Journal of Stroke 2014; 9(6): 824-8.

Chong JSX, Liu S, Loke YM, Hilal S, Ikram MK, Xu X*, et al.* Influence of cerebrovascular disease on brain networks in prodromal and clinical Alzheimer’s disease. Brain 2017; 140(11): 3012-22.

Fox MD, Corbetta M, Snyder AZ, Vincent JL, Raichle ME. Spontaneous neuronal activity distinguishes human dorsal and ventral attention systems. Proceedings of the National Academy of Sciences 2006; 103(26): 10046.

Greicius MD, Krasnow B, Reiss AL, Menon V. Functional connectivity in the resting brain: A network analysis of the default mode hypothesis. Proceedings of the National Academy of Sciences 2003; 100(1): 253.

Koechlin E, Basso G, Pietrini P, Panzer S, Grafman J. The role of the anterior prefrontal cortex in human cognition. Nature 1999; 399(6732): 148-51.

Seeley WW, Menon V, Schatzberg AF, Keller J, Glover GH, Kenna H*, et al.* Dissociable intrinsic connectivity networks for salience processing and executive control. The Journal of Neuroscience 2007; 27(9): 2349.

Sridharan D, Levitin DJ, Menon V. A critical role for the right fronto-insular cortex in switching between central-executive and default-mode networks. Proceedings of the National Academy of Sciences 2008; 105(34): 12569.

Vincent JL, Kahn I, Snyder AZ, Raichle ME, Buckner RL. Evidence for a frontoparietal control system revealed by intrinsic functional connectivity. J Neurophysiol 2008; 100(6): 3328-42.

Vipin A, Loke YM, Liu S, Hilal S, Shim HY, Xu X*, et al.* Cerebrovascular disease influences functional and structural network connectivity in patients with amnestic mild cognitive impairment and Alzheimer’s disease. Alzheimer's Research & Therapy 2018; 10(1): 82.

Zielinski BA, Gennatas ED, Zhou J, Seeley WW. Network-level structural covariance in the developing brain. Proceedings of the National Academy of Sciences 2010; 107(42): 18191.
